# Expression of the endocannabinoid system and response to cannabinoid components by the human fetal testis

**DOI:** 10.1186/s12916-023-02916-5

**Published:** 2023-07-11

**Authors:** J. Dochez-Arnault, C. Desdoits-Lethimonier, I. Matias, B. Evrard, M. Lagarrigue, M. Toupin, A. Lardenois, F. Chalmel, S. Mazaud-Guittot, N. Dejucq-Rainsford, A. Gely-Pernot

**Affiliations:** 1 Univ Rennes, Inserm (Institut National de La Santé Et de La Recherche Médicale), EHESP, Irset (Institut de Recherche en Santé, Environnement Et Travail) - UMR_S 1085 (Institut de Recherche en Santé, environnement et travail), 9 Avenue du Professeur Léon Bernard, 35000 Rennes, CEDEX, France; 2 Neurocentre Magendie - Inserm, U1215 Bordeaux, France

**Keywords:** Endocannabinoids, CBD, THC, Human fetal exposure, Endocrine disruptors, Testis, Germ cell development and cancer, Environmental factors

## Abstract

**Background:**

Cannabis consumption by pregnant women continues to increase worldwide, raising concerns about adverse effects on fetal growth and deleterious impacts on the newborn, in connection with evidence of placental transfer of cannabis compound. Cannabis action is mediated by the endocannabinoid system (ECS), which expression is well established in the brain but unknown in the developing testis. The fetal testis, whose endocrine function orchestrates the masculinization of many distant organs, is particularly sensitive to disruption by xenobiotics. In this context, we aimed to determine whether cannabis exposure has the potential to directly impact the human fetal testis.

**Methods:**

We determined the expression of components of the ECS in the human fetal testis from 6 to 17 developmental weeks and assessed the direct effects of phytocannabinoids Δ9-trans-tetrahydrocannabinol (THC) and cannabidiol (CBD) on the testis morphology and cell functions ex vivo.

**Results:**

We demonstrate the presence in the human fetal testis of two key endocannabinoids, 2-arachidonylglycerol (2-AG) and to a lower level anandamide (AEA), as well as a range of enzymes and receptors for the ECS. Ex vivo exposure of first trimester testes to CBD, THC, or CBD/THC [ratio 1:1] at 10^−7^ to 10^−5^ M altered testosterone secretion by Leydig cells, AMH secretion by Sertoli cells, and impacted testicular cell proliferation and viability as early as 72 h post-exposure. Transcriptomic analysis on 72 h-exposed fetal testis explants revealed 187 differentially expressed genes (DEGs), including genes involved in steroid synthesis and toxic substance response. Depending on the molecules and testis age, highly deleterious effects of phytocannabinoid exposure were observed on testis tissue after 14 days, including Sertoli and germ cell death.

**Conclusions:**

Our study is the first to evidence the presence of the ECS in the human fetal testis and to highlight the potential adverse effect of cannabis consumption by pregnant women onto the development of the male gonad.

**Supplementary Information:**

The online version contains supplementary material available at 10.1186/s12916-023-02916-5.

## Background

Cannabis is the most widely used drug in the world. It is also the commonest recreational drug consumed by pregnant women. In western countries, up to 20% of pregnant women are exposed to cannabis during pregnancy [1]. In the USA, while fetal exposure to tobacco decreases, cannabis use during pregnancy rises along with the enhanced perception that there are no risks with cannabis consumption during pregnancy [2]. Thus, an increase of cannabis use by 3.4–7% of pregnant women in the USA has been reported during this last decade [3]. However, data on pregnant women consumption remain rare and the use of self-reported questionnaires likely underestimates real exposures. Indeed, phytocannabinoid compounds have been evidenced in 5.3% of newborn meconiums, whereas only 1.7% of the mothers self-reported cannabis use [4]. Cannabis components and their metabolites are well known to cross the placenta barrier [5]. Use of cannabis during pregnancy can induce negative birth outcomes, such as reduced weight, increased risk of prematurity, cognitive deficits, and behavioral and neurocognitive impairment. To date, the effect of this consumption on the human genital tract is unknown [6–9].

The main chemicals present in recreational cannabis are Δ9-tetrahydrocannabinol (THC), which is responsible for the psychoactive effects of marijuana, and cannabidiol (CBD). The presence of THC in fetal blood after maternal ingestion and THC and CBD in meconium between 0 and 48 h after delivery indicate these two molecules cross the placenta [10–13]. THC and CBD act on specific cannabinoid receptors called CB1 and CB2. Both of these receptors also respond to naturally synthesized cannabinoids, namely anandamide (AEA) and 2-arachidonyl-glycerol (2-AG), so-called endocannabinoids. In addition to the endocannabinoids and their receptors, this endocannabinoid system (thereafter called ECS) comprises synthesis and degradation enzymes and transporters [14]. AEA and 2-AG, which both derive from membrane phospholipid precursors and arachidonic acid, are synthesized and metabolized by different pathways from the ECS. The ECS, which is sensitive to phytocannabinoids, plays a fundamental physiological role in the brain and peripheral tissues, as well as in many pathological conditions [14]. ECS has been involved in reproductive function at both the central and gonadal levels in human and animal models [15, 16]. Although cannabinoid receptors have been described in the mouse fetal testis [17], there is no description of the ECS in the human fetal testis. To date, the potential effect of cannabis exposure on the developing testis is unknown. We recently showed that the endocannabinoid system is present in the adult human testis [18]. In young men, several studies have demonstrated an association between cannabis consumption and testicular germ cell tumors (TGCTs) development [19–21], which could reflect the sensitivity of testicular cells to cannabis compounds.

In this context, we aimed at investigating the expression and localization of the ECS components in the human fetal testis and determining whether phytocannabinoids can directly affect fetal testicular cells, in order to unveil a potential implication of cannabis exposure in utero in impaired testis development and subsequent deleterious consequences, including testis cancer. Indeed, the testicular dysgenesis syndrome hypothesis implies that the alteration of the development and functions of any type of fetal testicular cell can have a negative impact on other distant cells and tissues [22, 23]. This is especially true in the case of impaired steroidogenesis by Leydig cells, since testosterone is key for fetal testis development and other organ masculinization. [22, 23]. In this study, we demonstrate that cannabinoid receptors CB1 and CB2 as well as endocannabinoid synthetizing and degrading enzymes are present in the human fetal testis. Moreover, our data uncover deleterious effects of direct exposures of THC and CBD onto the developing human testis and its endocrine functions ex vivo.

## Methods

### Chemicals

CBD (Axon Medchem, Netherlands) was diluted in dimethylsulfoxide (DMSO) solution, while THC (Sigma-Aldrich; CAS number 1972–08-3, USA) was diluted in ethanol (EtOH) solution. The same amount of DMSO, EtOH, and a mixture of DMSO and EtOH were used as controls for CBD, THC, and THC/CBD mixture, respectively.

### Human fetal testis collection and ethic approval

First trimester (7–12 developmental weeks (DW), i.e., 9–14 weeks from last menstrual) human fetuses were obtained from pregnant women following legally induced abortion performed in Rennes University Hospital (France). None of the pregnancy terminations were due to fetal abnormalities. All women received information about the project and gave informed written consent in accordance with national guidelines enacted by the French Agency for Biomedical Research (Agence de la Biomédecine, #PFS09-011). The local ethic committee of Rennes hospital (advice #11–48) approved the protocol. The aspiration products were placed at 4 °C after collection and rapidly processed. The testes were recovered from the aspiration products under a binocular microscope (Olympus SZX7, Lille, France) and immediately placed in cold phosphate-buffered saline (PBS) solution.

### Organotypic cultures of fetal testis

The fetal testes were cut into explants of 1mm^3^ and placed in cell culture inserts (0.4 µm pores, Falcon, Becton–Dickinson, Le Pont de Claix, France) put in 24-well companion culture plates (Falcon, Becton–Dickinson) according to a standardized protocol previously described [24, 25]. On each insert, 3 or 4 testicular explants were placed and cultivated in 350 µl of phenol red-free medium 199 (Invitrogen Life Technologies, Saint-Aubin, France) supplemented with 50 µg/mL gentamicin (Sigma-Aldrich, Saint-Quentin, France) and 2.5 µg/mL Fungizone (Sigma-Aldrich). As determined in previous experiments, human chorionic gonadotropin (hCG, Sigma-Aldrich) was added to a final concentration of 0.1 IU/mL in order to maintain steroidogenic responsiveness of the testicular tissue. The cultures were incubated at 37 °C for 96 h in a humidified atmosphere of 95% air and 5% CO_2_. The medium was removed every 24 h and divided into 2 aliquots that were immediately snap-frozen on dry ice and stored at − 80 °C. Explants were cultured in medium 199 supplemented with hCG for the first 24 h of culture (D0) without any drug, in order to define a baseline for hormone production in each well. This allows normalization of the explant secretory capacity when the drugs are added [24]. After D0, the explants were exposed to treatments for 3 days by adding CBD and/or THC at concentrations of 10^−5^ M, 10^−6^ M, or 10^−7^ M to the medium, or to dimethylsulfoxyde (DMSO, Sigma-Aldrich) and/or ethanol, the solvents of CBD and THC, respectively. Medium was changed every 24 h. The range of concentrations of THC and CBD was chosen based on peak plasma concentrations (Cmax) in adults, which differ depending on the mode of administration of cannabis. When smoked, the most common consumption method, THC Cmax, usually reaches 10^−7^ M [26]. Phytocannabinoid pharmacokinetics may differ from occasional to heavy users and according to the user’s age [27]. Since only few studies assessed the quantitative relation between mother consumption and the distribution of phytocannabinoids in the placental and fetal compartment, we decided to study a large range of concentration, from 10^−7^ M to 10^−5^ M.

For the 2-week-long culture, insulin, transferrin, and selenium (ITS, Corning, Ref 354352) were added to the previously described culture medium, as we found it entitled better preservation of fetal testis morphology and functions in longer culture. In this case, explants were cultured for the first 24 h of culture without drugs and then with either control, CBD 10^−5^ M, THC 10^−5^ M, CBD/THC 10^−6^ M, or CBD/THC 10^−5^ M. The medium was changed every 48 h and kept frozen at − 80 °C at day 4 (D4), D8 and D14 for subsequent analysis.

### MALDI imaging of 2-AG

A 11 DW human fetal testis was embedded in carboxymethylcellulose (M-1 embedding matrix, Thermo Scientific) then frozen on dry ice and stored at − 80 °C. Sections 10-µm-thick were prepared with a CM 3050S cryostat (Leica) at − 17 °C and thaw-mounted onto a glass slide coated with indium tin oxide (Bruker). The MALDI matrix solution was composed of 2,5-dihydroxybenzoic acid (DHB) at 50 mg.mL^−1^ in methanol/water/trifluoroacetic acid (70/30/0.1). An HTX M5 sprayer (HTXImaging) was used to deposit the DHB solution on the slide by performing 8 passes at a spray temperature of 75 °C and a flow rate of 0.1 mL.min^−1^. The tissue section was analyzed in positive ion mode on a 7 T MALDI-FT-ICR SolariX XR mass spectrometer (Bruker). Spectra were acquired in the m/z 150–900 mass range with a resolving power of 130,000 at m/z 400 and a lateral resolution of 10 µm. Every spectrum was calibrated with internal lock masses. MALDI images were reconstructed and normalized on root mean square using the fleximaging 5.0 software.

### Endocannabinoid measurements in fetal testes

Testes were removed, flash frozen, and stored at − 80 °C until analysis. Measurements of anandamide (AEA) and 2-arachidonoylglycerol (2-AG) were carried out as previously described (Lourenço et al. 2011). Briefly, tissues were homogenized and extracted by liquid–liquid extraction with chloroform/methanol/Tris–HCl 50 mM, pH 7.5 (2:1:1, vol/vol) containing internal deuterated standards (AEA-d4, PEA-d4, OEA-d4, and 2-AG-d5). Phase separation was facilitated by centrifugation at 10,000* g* for 2 min. The lipid-containing organic phase was concentrated on an N2 stream evaporator, while the protein-containing aqueous phase was treated with trichloroacetic acid (TCA) in order to precipitate the protein. Protein amounts were determined by the Lowry method using the BCA protein Pierce (ThermoScientific, CA) assay with bovine serum albumin (BSA) as standards. The dried lipid extracts were then subjected to isotope-dilution liquid chromatography-chemical ionization-tandem mass spectrometric analysis. Mass spectral analyses were performed on a TSQ Quantum Access triple quadrupole instrument (Thermo-Finnigan) equipped with a APCI source (atmospheric pressure chemical ionization) and operating in positive ion mode. The TSQ Quantum Access triple quadrupole instrument was used in conjunction with a Surveyor LC Pump Plus (Supelco C18 Discovery Analytical column) and cooled autosampler. The amounts of anandamide and 2-AG were determined by isotope-dilution using a calibration curve and expressed as pmol by mg of proteins.

### Hormone measurements in fetal testes

Hormone levels were measured in the culture medium of testis explants at D0 and D3 for short-, and at D0, D4, D8, and D14 for long-term culture experiments. Each sample was assayed in duplicate for all hormone measurements. Testosterone levels were assayed with a specific radioimmunoassay (RIA) kit (Beckman Coulter, Ref IM1119), according to the kit manufacturer’s instructions. Insulin-like factor 3 (INSL3) levels was measured using a specific RIA kit according to the kit manufacturer’s instructions (Phoenix, Ref RK-035–27). Anti-Müllerian Hormone (AMH) levels were assayed by Enzyme-Linked Immunosorbent Assay (ELISA) kit (Beckman Coulter, Ref A79765). The hormone assay values are all expressed as fold change compared to the mean of their respective control, after a normalization of hormone production with D0 values for each sample.

### Immunohistochemistry and cell counting

Immunohistochemistry was performed on Bouin solution-fixed, paraffin-embedded, 5 µm-thick sections of testis explants. Each fifth section was used for immunohistochemical analysis with cell-specific labeling, as previously described [24, 25, 28]. Following dewaxing and rehydration, antigen retrieval was performed for all immunostaining by treating the sections with 10 mM citrate buffer (pH 6.0) at 80 °C for 45 min or Tris-Ethylenediaminetetraacetic acid (EDTA buffer (pH 9.0) at 80 °C for 30 min before cooling them at room temperature (RT). Non-specific sites were then blocked with a 10% BSA or chicken serum treatment for 1 h at RT and sections were incubated overnight at 4 °C with the primary antibody diluted in Dako antibody diluent (Dako, Ref S3022) or PBS-Tween 0.01%.

Leydig cells were labelled with a rabbit primary antibody directed against the cytochrome P450, family 11, subfamily A, polypeptide 1 (CYP11A1) (1:250, Sigma-Aldrich, Ref HPA016436). Germ cells were stained using LIN28 rabbit primary antibody (1:800, Abcam, Ref Ab462020). Sertoli cells were labelled using AMH goat primary antibody (1:200, Santa-Cruz, Ref sc6886). Apoptotic and proliferating cells were stained with a polyclonal rabbit primary antibody directed against cleaved caspase-3 (1:100; Cell Signaling Technology, Ref 9661), and a monoclonal mouse primary antibody directed against KI-67 (1:100; Dako, Ref M7240), respectively. The appropriate biotinylated goat anti-mouse (Dako, Ref E0433) and anti-rabbit (Vector, Ref BA-1000), and rabbit anti-goat (Dako, Ref E0466) antibodies were used at 1:500 as previously described [29]. Signal corresponding to the secondary antibody was amplified by an incubation with an avidin and biotinylated enzyme complex (ABC Vectastain Elite Kit, Vector Laboratories). Sections were developed using 3,3’-diaminobenzidine tetrahydrochloride (DAB, Sigma-Aldrich) and then counterstained with Masson’s Hemalun and Lithum Carbonate before dehydration. Between all steps (except between blocking of non-specific sites and the first antibody incubation), sections were washed using phosphate buffer saline (PBS) with 0,1% of Tween 20 detergent (Sigma-Aldrich). The number of LIN28-positive germ cells was determined by counting the stained cells in 8–12 randomly selected histological explant sections of each condition, using a light microscope (Bh2 Olympus Microscope) coupled to Mercator Expert Software (Explora NOVA). For the 3-day cultures, apoptotic cleaved caspase 3-positive and proliferative KI67-positive cells were counted in the testis cords (including germ and Sertoli cells) and interstitial tissue (including Leydig cells) in 8–12 randomly selected histological explant sections of each condition, giving so-called “intracordonal” and “extracordonal” counting values. The surface area of each section was measured and data presented as cell number per surface area unit.

In long-term cultures, relative Leydig and Sertoli cells, and apoptotic cell area were estimated by relating the surface occupied by the CYP11A1-positive Leydig cells, AMH-positive Sertoli cells, and cleaved caspase-3-positive cells respectively onto the total surface of 8–12 randomly selected histological explant sections. For this purpose, we measured the surface occupied by each investigated cell type labelled in dark brown by DAB and related it to the surface of the corresponding explant using ImageJ software (US National Institutes of Health, Bethesda, MD, USA).

### In situ hybridization (RNAscope)

RNAscope was performed on 5 μm formalin-fixed, paraffin-embedded tissue sections from 6 different donors of 7–14 DW using RNAscope 2.5 HD-Red kit (Advanced Cell Diagnostics; ACD) according to the manufacturer’s instructions with some modifications. Briefly, the slides were dried 1 h at 60 °C, deparaffinized, and pretreated for 10 min at RT with H_2_O_2_. For target retrieval, sections were boiled with the manufacturer’s target retrieval buffer (ACD Bio-techne) for 15 min at 90–100 °C and then washed twice in distilled water and once then 100% EtOH. To increase target accessibility, protease digestion was then carried out by incubating sections with protease plus solution (ACD Bio-techne) diluted 1:5 in PBS-DEPC at 40 °C for 30 min (HybEZ Oven, ACD Bio-techne). Sections were finally washed with distilled water before proceeding to probe hybridization. Sections were incubated with the *CNR1* (Bio-techne, ref 591521) and *CNR2* (Bio-techne, ref 596021) gene’s probes for 2 h at 40 °C. Positive (Polr2A; Bio-techne, ref 310451) and negative (DapB; Bio-techne, ref 310043) controls, developed by ACD Bio-techne, were also tested on our tissue sections to validate tissue quality and technique specificity. The slides were washed 2 times during 2 min in 1X wash buffer at RT, and the hybridized signals were then amplified by six consecutive signal amplification steps (Hybridize Amp 1–6, ACD Bio-techne) according to the manufacturer’s instructions, with the following modification: sections were incubated with Hybridize Amp 5 for 60 min at RT and washed twice 2 min each in 1X wash buffer (ACD Bio-techne) at RT after each signal amplification step. For signal detection, sections were incubated for 10 min at RT in red solution (RNAScope 2.5 HD Assay–RED, ACD Bio-techne) and rinsed in distilled water for 2 min.

### Immunofluorescence

Sections stained for RNAscope were blocked with PBS-BSA at 10% for 30 min at RT and incubated overnight at 4 °C with primary antibody. Leydig cells were labelled with a rabbit primary antibody directed against CYP11A1 (1:100, Sigma-Aldrich, Ref HPA016436). Germ cells were stained with a LIN28 rabbit primary antibody (1:500, Abcam, Ref Ab46020). Sertoli cells were labelled with an AMH goat primary antibody (1/100, Santa-Cruz, Ref sc6886). Serial frozen sections from MALDI imaging were rehydrated in PBS, submitted to antigen retrieval with citrate buffer as described above, and processed for fluorescent CYP11A1 and AMH double immunostaining. The appropriate AF488 chicken anti-rabbit (Invitrogen, Ref A21441) or AF488 Donkey anti-goat secondary antibody (Invitrogen, Ref A11055) were used at 1:500 for 2 h at RT. PBS with 0.1% of Tween 20 detergent (Sigma-Aldrich) was used for washing steps. Slides were then mounted using Invitrogen Prolong Gold Antifade Mountant with DAPI (Invitrogen, P36935).

### RT-qPCR

Total RNA was extracted using RNA/DNA extraction kit (Qiagen, Germany). After extraction, RNA was precipitated, washed, and diluted in RNA-free water. RNA quantity was assessed using a NanoDrop™ 8000 Spectrophotometer (Thermo Fisher Scientific), and RNA quality using a 2100 Bioanalyzer Instrument (Agilent Technologies, CA, USA). RT-qPCR was performed as described using iTaq Universal SYBR Green Supermix (Bio-Rad) and cDNA template in a CFX384 Touch Real-Time PCR Detection System (Bio-Rad). RPLP0 mRNAs were used as internal controls for normalization. Primers are listed in Table 1. Results calculated using the ΔΔCT method are presented as *n*-fold differences in target gene expression relative to reference gene and calibration sample.

**Table 1:** Primers used for qPCR experiments to characterize isoforms of endocannabinoid receptors

Target	Primer sequence (5′-3′)	Amplicon size	Annealing temperature
CNR1 (CB1)	F: TCAGTACGAAGACATCAAAGGTG	85pb	60 °C
	R: CTTCCCCTAAAGGAAGTTAAAGG		
CB1A	F: AGACATCAAAGGAGAATGAGGAG	113pb	60 °C
	F: AGACATCAAAGGAGAATGAGGAG		
CB1B	F: AGACATCAAAGGAGAATGAGGAG	84pb	64 °C
	R: AATGTTCACCTGGTCTGCTG		
CB2A	F: GATTATGCCAGCCAGATGC	77pb	64 °C
	R: GCTCGGTGAGTGAGAGGTG		

### Statistical analyses

The effect on testosterone secretion of CBD and/or THC treatments at various concentrations versus control was assessed using Freidman tests followed by the two-stage linear step-up procedure of Benjamini, Krieger, and Yekutieli. For cell counting, AMH, Inhibin B, and INSL3 measurements, the control conditions, and CBD and/or THC 10^−5^ M treatments were compared two by two using Wilcoxon tests. For RNA-seq data analysis, mRNA expression between two ages was assessed using Kruskal–Wallis tests followed by the two-stage linear step-up procedure of Benjamini, Krieger, and Yekutieli. For long culture treatment, Friedman test comparisons was used to measure difference between control and treated conditions, followed by two-stage linear step-up procedure of Benjamini, Krieger, and Yekutieli. Statistical significance threshold was set at 0.05. Data are expressed as mean ± SEM.

### RNA-seq experiments and data analysis

RNA-seq raw data were mined from our previously published dataset [30] to study the dynamic of gene expression of endocannabinoids synthetizing and degrading enzymes.

### BRB-seq experiments and data analysis

#### Preparation of the sample

Total RNA was extracted from 8 to 10 (*n* = 10) and 10 to 12 DW fetal testis explants (*n* = 10) after 72 h exposure to CBD, THC, or CBD/THC at 10^−5^ M and their respective controls, as described above.

#### Library preparation and sequencing

3’BRB-seq experiments were performed as previously described [31–33]. Briefly, RNAs were distributed onto two 96-well plates. A first step of reverse transcription and template switching reactions was performed using 4 µL total RNA at 2.5 ng/µL and sample-specific barcoded oligo-dT. Then, cDNAs from each plate were pooled together and purified and double-strand (ds) cDNAs were generated by PCR. The two corresponding sequencing libraries were next built by tagmentation using 50 ng of ds cDNA with the Illumina Nextera XT Kit (Illumina, #FC-131–1024) following the manufacturer’s recommendations. The resulting library was finally sequenced on a NovaSeq sequencer as Paired-End 100 base reads by the IntegraGen Company (https://integragen.com/fr/). Image analysis and base calling were performed using RTA 2.7.7 and bcl2fastq 2.17.1.14. Adapter dimer reads were removed using DimerRemover (https://sourceforge.net/projects/dimerremover/).

#### Data preprocessing and normalization

A phred quality score higher than 10 was required for the first reads (R1, 16 bases). Among these, the first 6 bases correspond to the unique sample-specific barcode needed to demultiplex the sequencing data, while the following 10 bases correspond to a unique molecular identifier (UMI) used for quantification purposes. The second reads (R2) were aligned to the human reference transcriptome from the UCSC website using BWA version 0.7.4.4 with the parameter “ − l 24”. Reads mapping to several positions in the genome were filtered out from the analysis. After quality control and data preprocessing, a gene count matrix was generated by counting the number of unique UMIs associated with each gene in lines for each sample in columns. The UMI matrix was further normalized with the regularized log (rlog) transformation package implemented in the DeSeq2 package [34]. Raw and preprocessed data will be deposit at the GEO repository. The GEO accession number is GSE223827 [35].

#### Differential gene expression analysis

Principal component analysis (PCA) and Uniform *Manifold* Approximation and Projection (UMAP) were performed with the FactoMineR [36] and umap packages implemented in R v4.1.1. Differentially expressed genes (DEGs) were identified by comparing each compound (CBD, THC, CBD + THC) to their corresponding control samples at each developmental stage (10–12GW and 12–14GW). For each comparison, different filtration steps were applied: (i) the median gene expression value (= 0.0) of all the samples was used as a background cut-off; (ii) a foldchange cut-off of at least 1.3; and (iii) a statistical filtration with a paired samples *t*-test and a *p*-value cut-off of 0.05 adjusted with the Benjamini & Hochberg method [37]. The resulting transcriptomic signatures was deposited at the TOXsIgN repository (https://toxsign.genouest.org/) [38].

#### Clustering and functional analysis

The resulting lists of DEGs were clustered into distinct gene expression patterns with the mclust algorithm [39]. A Gene Ontology term enrichment analysis was performed with the Annotation Mapping Expression and Network (AMEN) [40]. A specific annotation term was considered significantly enriched in a given gene expression pattern when the false discovery rate (FDR)-adjusted *p*-value (Fisher’s exact probability) was ≤ 0.05 and the number of associated genes was ≥ 3.

## Results

### The endocannabinoid system is dynamically regulated in the fetal testis at 6 to 17 weeks of development

**Fig. 1:**
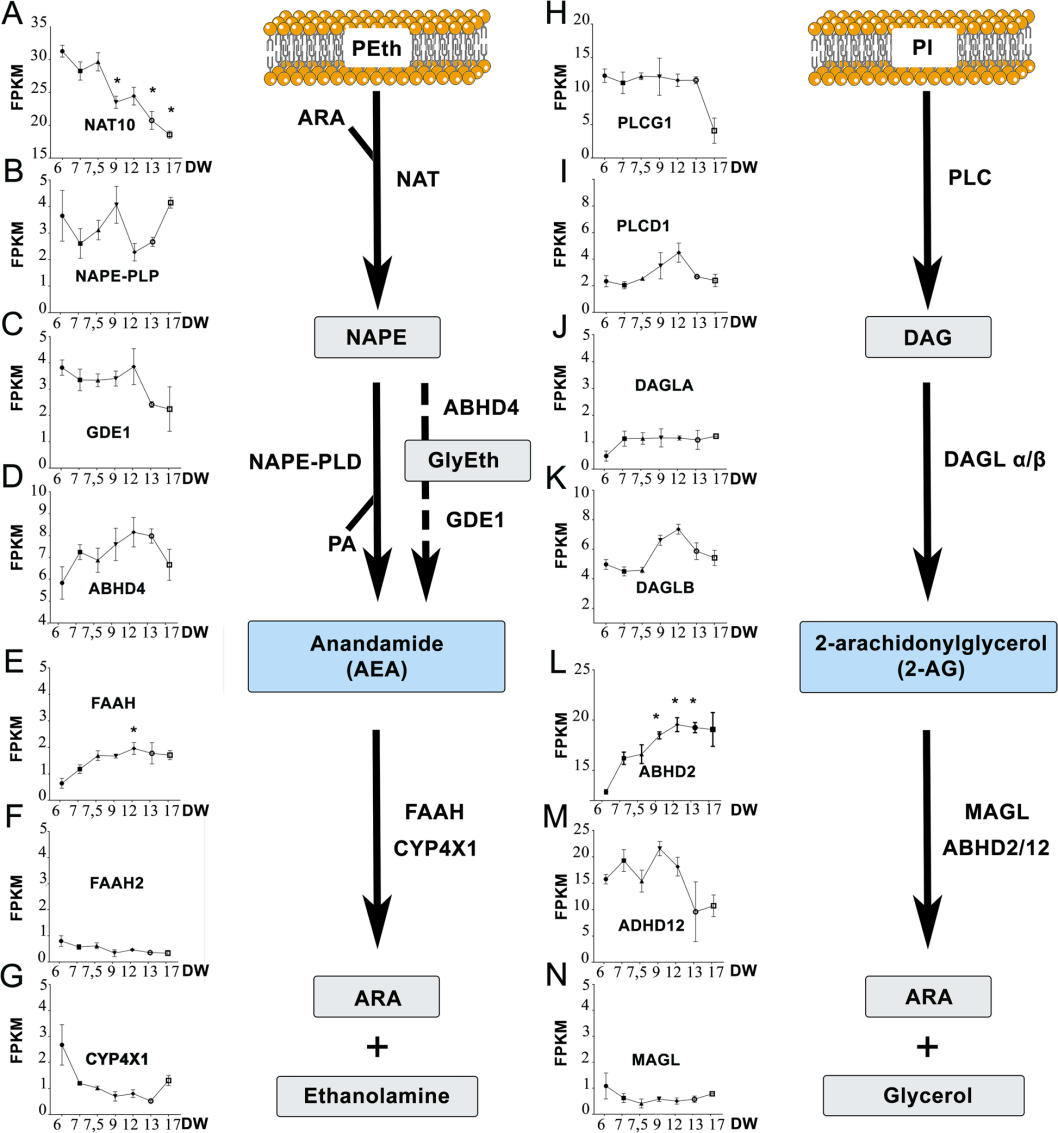
Expression of ECS enzymes in human fetal testis. RNA-seq quantification of the expression of transcripts encoding AEA synthetizing (**A–D**) and degrading enzymes (**E–G**), and 2-AG synthetizing (**H–K**) and degrading (**L–N**) enzymes in bulk embryonic human testis from 6 to 17 developmental weeks (DW). Transcript amount is expressed in Fragments Per Kilobase Million (FPKM). Data presented as means + / − SEM. An ANOVA test was performed followed by a Kruskal–Wallis test to compare the transcript at 6 DW with other ages (*, *p* < 0.05) and a two-stage linear step-up procedure (Benjamini, Krieger and Yekutieli). A schematic representation of endocannabinoid synthesis and degradation pathways is shown. Endocannabinoids derive from membrane phospholipid precursors, phosphatidylethanolamine (PEth), phosphatidylinositol (PI), and arachidonic acid (ARA). The major synthetizing enzymes of AEA are N-acetyltransferase 10 (NAT10) and N-acyl-phosphatidylethanolamine-hydrolyzing phospholipase D (NAPE-PLD). The alternative pathway of AEA synthesis involves Abhydrolase domain-containing protein 4 (ABHD4) to produce intermediate molecules, glycerophosphoacylethanolamides (GlyEth), and glycerophosphodiester phosphodiesterase 1 (GDE1). The synthetizing enzymes of 2-AG are phospholipase C-gamma-1 (PLCG1), phospholipase C-delta-1 (PLCD1), diacylglycerol lipase-alpha (DAGLA) and -beta (DAGLB). The degrading enzymes of AEA are fatty acid amide hydrolase (FAAH and FAAH2) and cytochrome P450 4X1 (CYP4X1). The degrading enzymes of 2-AG are Abhydrolase domain-containing protein 2 and 12 (ABHD2, and 12) and monoglyceride lipase (MAGL). The degradation of endocannabinoids induce the production of ARA and ethanolamine or glycerol

**Fig. 2:**
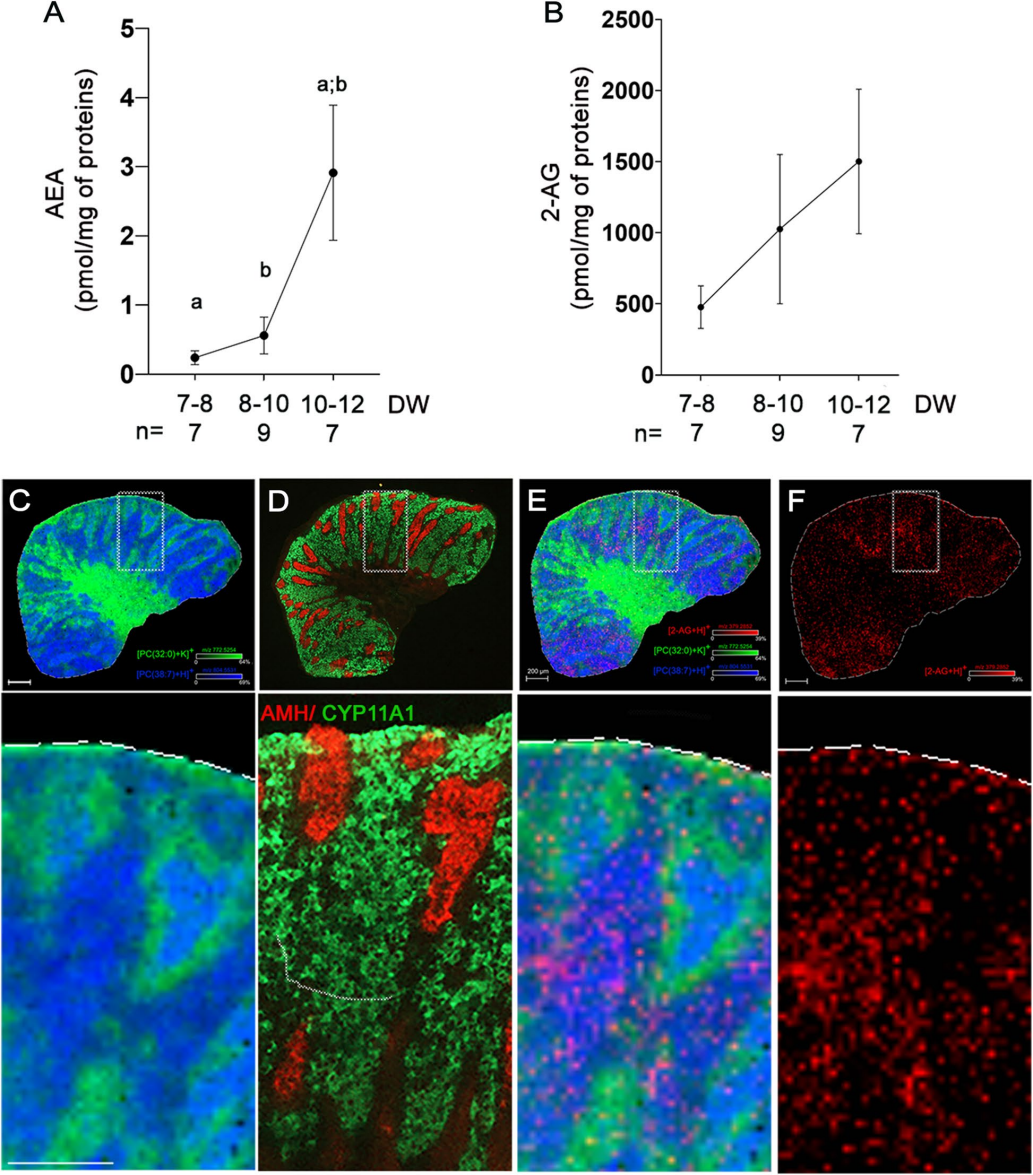
Endocannabinoid synthesis in human fetal testis. AEA (**A**) and 2-AG (**B**) concentrations measured by mass spectrometry in human fetal testes at 7–8 DW (*n* = 7), 8–10 DW (*n* = 9), and 10–12 DW (*n* = 7). Data in pmol per mg of proteins in the tissue are presented as mean + / − SEM. ANOVA test was performed followed by Kruskal–Wallis test for pair comparisons (a and b: *p* < 0.01). (**C**) MALDI images showing the distribution of two prototypic lipids in the fetal testis: [PC(32:0) + K]^+^ (m/z 772.5254 in green) in testis cords, surrounding cells, and tunica albuginea; [PC(38:7) + H]^+^ (m/z 804.5531 in dark blue) in interstitial tissue and cords in 11 DW testis. Lateral resolution = 10 µm, m/z window = 3 ppm, scale bars = 200 µm. PC = phosphatidylcholine. (**D**) Immunohistochemistry on adjacent testis sections using Leydig cell marker CYP11A1 (green) and Sertoli cell marker AMH (red) antibodies. Detection of 2-AG (m/z 379.2582 in red) (**E**) merged with [PC(38:7) + H]^+^ and [PC(32:0) + K]^+^ or (**F**) alone. Scale bar = 200 µm

The ECS comprises synthesis and degradation enzymes responsible for the regulation of endocannabinoids, AEA and 2-AG (Fig. 1) and their receptors CB1 and CB2. The expression of synthetizing/degrading enzymes of AEA (Fig. 1A–G) and 2-AG (Fig. 1H–N) was investigated using a previously published RNA-seq dataset [30] arising from the analysis of the genome of 6 to 17 DW human testis bulk RNA. During the gestational period spanned, various dynamics of expression were observed for the different enzymes of the ECS. For synthetizing enzymes, the expression of transcripts encoding *NAT10* significantly decreased between 6 and 17 DW, whereas the expression of transcripts encoding *NAPE-PLD, GDE1 ABHD4, PLGC1*, *PLCD1 DAGLA*, and *DAGLB*, did not vary significantly (Fig. 1A–D, H–K). For degrading enzymes, the expression of transcripts encoding *FAAH* and *ABHD2* significantly increased, while no significant changes were found for *FAAH2, CYP4X1, ABHD12,* and *MAGL* (Fig. 1E–G, L–N).

The presence of the two key endocannabinoids, AEA and 2-AG, was investigated by HPLC in human testis of 7–8, 8–10, and 10–12 DW (*n* = 7 to 9 for each age). The low amounts of AEA, ranging between 0.239 + / − 0.0996 and 2.913 + / − 0.977 pmol per mg of protein (Fig. 2A), significantly increased between 7–8 and 10–12 DW and 8–10/10–12 DW. In contrast, the fetal human testis contained high amounts of 2-AG, between 477.05 + / − 149.64 and 1502.10 + / − 508.20 pmol of 2-AG per mg of protein (Fig. 2B). The increase of 2-AG secretion during development did not reach statistical significance.

The intra-testicular localization of AEA and 2-AG was next investigated using MALDI imaging methodology. We first analyzed the distribution of two lipids displaying a typical intratesticular localization and compared it with that of Leydig cell marker CYP11A1 and Sertoli cell marker AMH using immunofluorescence on serial sections of 11 DW testis (Fig. 2C,D). As expected, PC (32:0) lipid (PC = phosphatidylcholine; m/z 772.5254 in green) was observed in testis cords as well as broadly distributed in the interstitial tissue of the hilum, while PC (38:7) lipid (m/z 804.5531 in dark blue) localized in CYP11A1-rich areas of the interstitial tissue and within the testis cords. Consequently, testis cords containing both lipid types appeared in clear blue. In agreement with the above HPLC result showing a low amount of AEA, this endocannabinoid could not be detected by MALDI imaging, as opposed to 2-AG, which was clearly present in the tissue and colocalized mainly with the PC(38:7) lipid, corresponding to Leydig cells area (Fig. 2E,F).

**Fig. 3:**
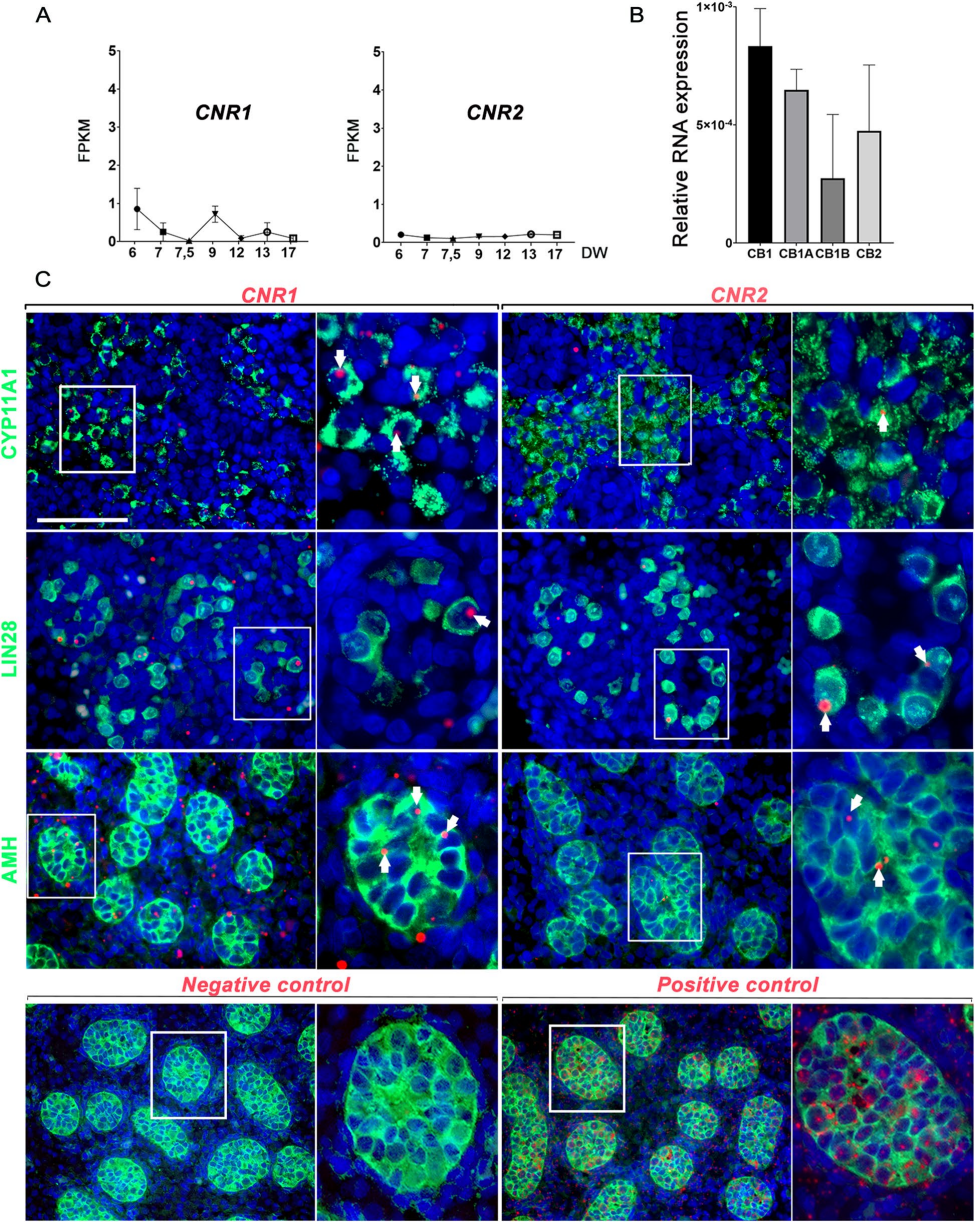
Cannabinoid receptors expression in human fetal testis. (**A**) RNA-seq quantification of transcripts coding for CB1 (*CNR1*) and CB2 *(CNR2*) in bulk embryonic human testis from 6 to 17 DW. Transcripts amount is expressed in Fragments Per Kilobase Million (FPKM). (**B**) Expression profiles of transcript isoforms coding for CB1, CB1A, CB1B, and CB2A. RT-qPCR was performed on testes from 3 human embryos at 7 DW. Histograms represent mean expression (relative to RPLP0) ± SEM. (**C**) Detection of *CNR1* and *CNR2* transcripts by RNAscope (red dots, some pointed by white arrows) on 10–12 DW human testis section co-immunostained with specific antibodies for Leydig cells (CYP11A1-positive cells), germ cells (LIN28-positive cells), and Sertoli cells (AMH-positive cells) in green. The right panel of each probe corresponds to an higher magnification. Scale bar = 70 and 30 µm for the higher magnification. The detection of dihydrodipicolinate reductase (dapB) and polymerase II transcripts was used as negative and positive control respectively on 10–12 DW human testis section co-immunostained with specific antibodies for Sertoli cells

The expression of CB1 (*CNR1*) and CB2 (*CNR2*) receptors mRNAs in the fetal testis was investigated using our RNA-seq dataset [30], as above (Fig. 3A) and confirmed in RT-qPCR on bulk testes of 7 DW (Fig. 3B). The expression of CB1 and CB2 receptor transcripts in the fetal human testis did not significantly vary in between 6 and 17 DW. *CNR1* and *CNR2* transcripts were further evidenced in situ by RNAscope in 10–12 DW testes (Fig. 3C). Dots corresponding to *CNR1* transcripts were more abundant than those corresponding to *CNR2*, in line with our RNA-seq and qPCR data. *CNR1* transcripts were mainly found in LIN28-positive germ cells and CYP11A1-positive Leydig cells, with possible low expression in AMH-positive Sertoli cells, while small dots corresponding to CNR2 were occasionally found in these three cell types. Despite several attempts, we could not reliably detect the expression of CB1 and CB2 using commercial antibodies on tissue slides in immunohistochemistry due to high unspecific staining.

### 72 h exposure of human fetal testis to phytocannabinoids ex vivo impacts the number of proliferating and apoptotic cells

To decipher the effect of phytocannabinoids at the cellular level, the density of KI67-positive proliferating cells and apoptotic cleaved caspase 3-positive cells was investigated in the two compartments of the fetal testis, i.e., extracordonal (containing Leydig cells, immune cells and fibroblasts), and intracordonal (containing Sertoli and germ cells) after 72 h of exposure of 10^−5^ M of each compounds.

**Fig. 4:**
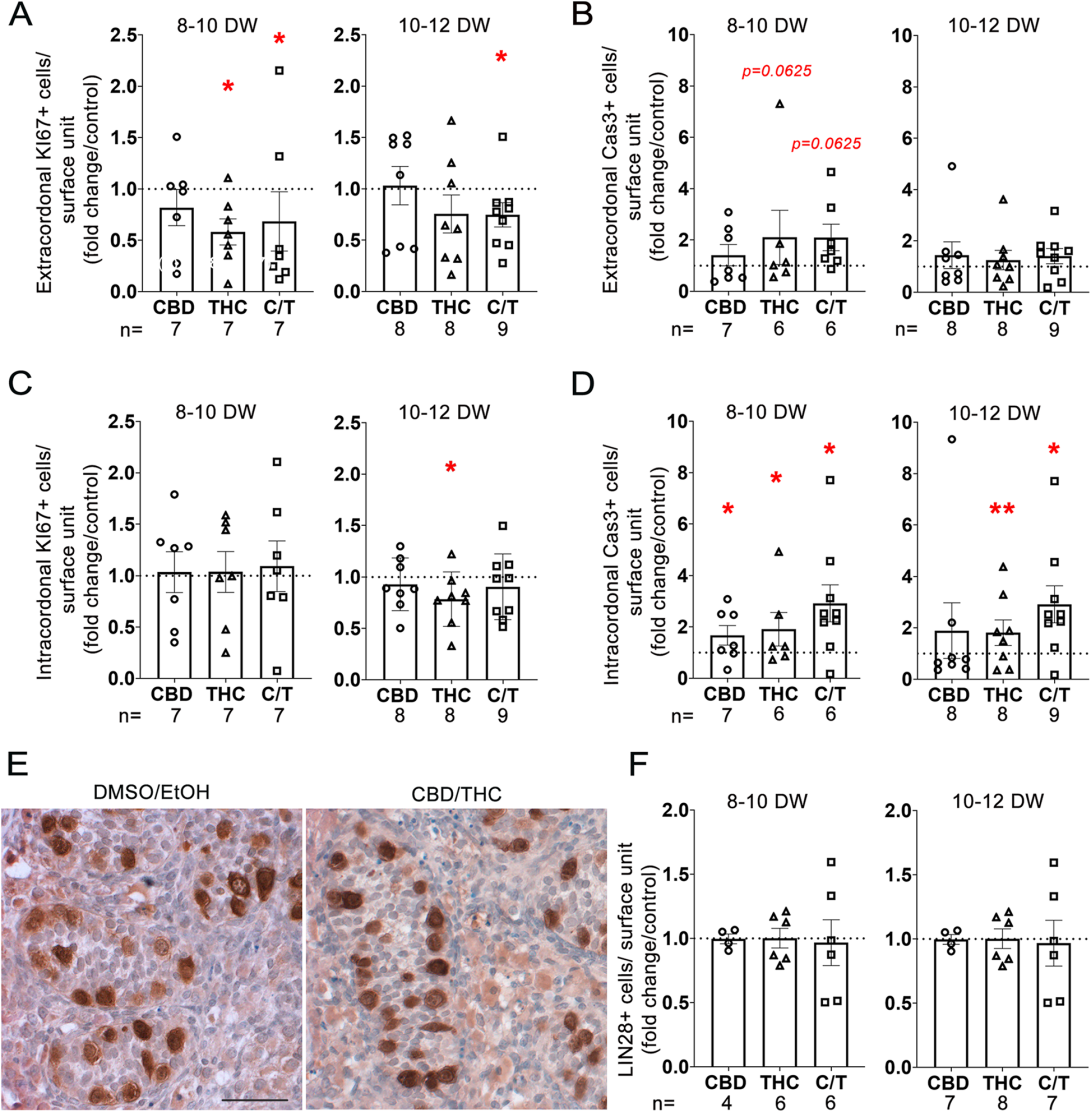
Effect of CBD and/or THC on proliferation, apoptosis, and germ cell numbers. Density of (**A**) proliferating cells (KI67 +) and (**B**) apoptotic cells (Cas3 +) in (**A**,**B**) extracordonal and (**C**,**D**) intracordonal tissue after CBD and/or THC treatment at 10^−5^ M. Results of cell quantification normalized by surface unit and expressed as fold from respective controls in 8–10 and 10–12 DW embryonic testes. Data presented as means + / − SEM. An ANOVA test was performed followed by a Wilcoxon test for pair comparisons (*, *p* < 0.05; **, *p* < 0.01). (**E**) Representative immunostaining of LIN28, a specific marker of germ cell in control (DMSO/EtOH) and CBD/THC at 10^−5^ M treated explants from a 10–12 DW human fetal testis. LIN28 appears dark brown (DAB staining) in the cytoplasm of germ cells within the intracordonal tissue. Sections were counterstained with hematoxylin (in blue). No modification of germ cell morphology was observed. (**F**) Density of LIN28 positive cells (LIN28 +) in intracordonal tissue after CBD and/or THC treatment at 10^−5^ M compared to respective controls in 8–10 and 10–12 DW embryonic testis. Scale bar = 50 µm

In the extracordonal compartment, the density of KI67-positive cells significantly decreased in 8–10 DW testis explants treated with THC or with CBD + THC compared to controls (*p* = 0.0469 for both) (Fig. 4A). In 10–12 DW testis explants, THC alone had no effect on the density of proliferating cells, whereas a decrease was observed when mixed with CBD (*p* = 0.0117). CBD alone did not affect the capacity of cells to proliferate in either 8–10 WD or 10–12 DW testes. CDB and/or THC did not increase the density of extracordonal apoptotic cells in either 8–10 or 10–12 DW testis explants, as compared with controls (Fig. 4B).

Within the cords, different patterns were observed. A significant decrease of the density of KI67-positive cells was observed exclusively for THC in 10–12 DW testis explants (*p* = 0.0391), whereas other conditions did not reach significance (Fig. 4C). In 8–10 DW testis explants, a significant increase in the density of cleaved caspase 3-positive intracordonal cells was counted for THC (*p* = 0.0313), CBD (*p* = 0.0234), and CBD/THC (*p* = 0.0313) (Fig. 4D). In 10–12 DW testis explants, THC and CBD/THC increased cell apoptosis (*p* = 0.0078 and *p* = 0.0117 respectively), whereas CBD had no significant effect.

We next assessed the impact of a 3-day treatment with 10^−5^ M of phytocannabinoids on the density of LIN28-positive germ cells in fetal testis cords (Fig. 4E, F). No significant change in the density of LIN28-positive germ cells could be measured at this early time point (Fig. 4F).

### 72 h exposure of human fetal testis to phytocannabinoids ex vivo decreases testosterone secretion by Leydig cells

**Fig. 5:**
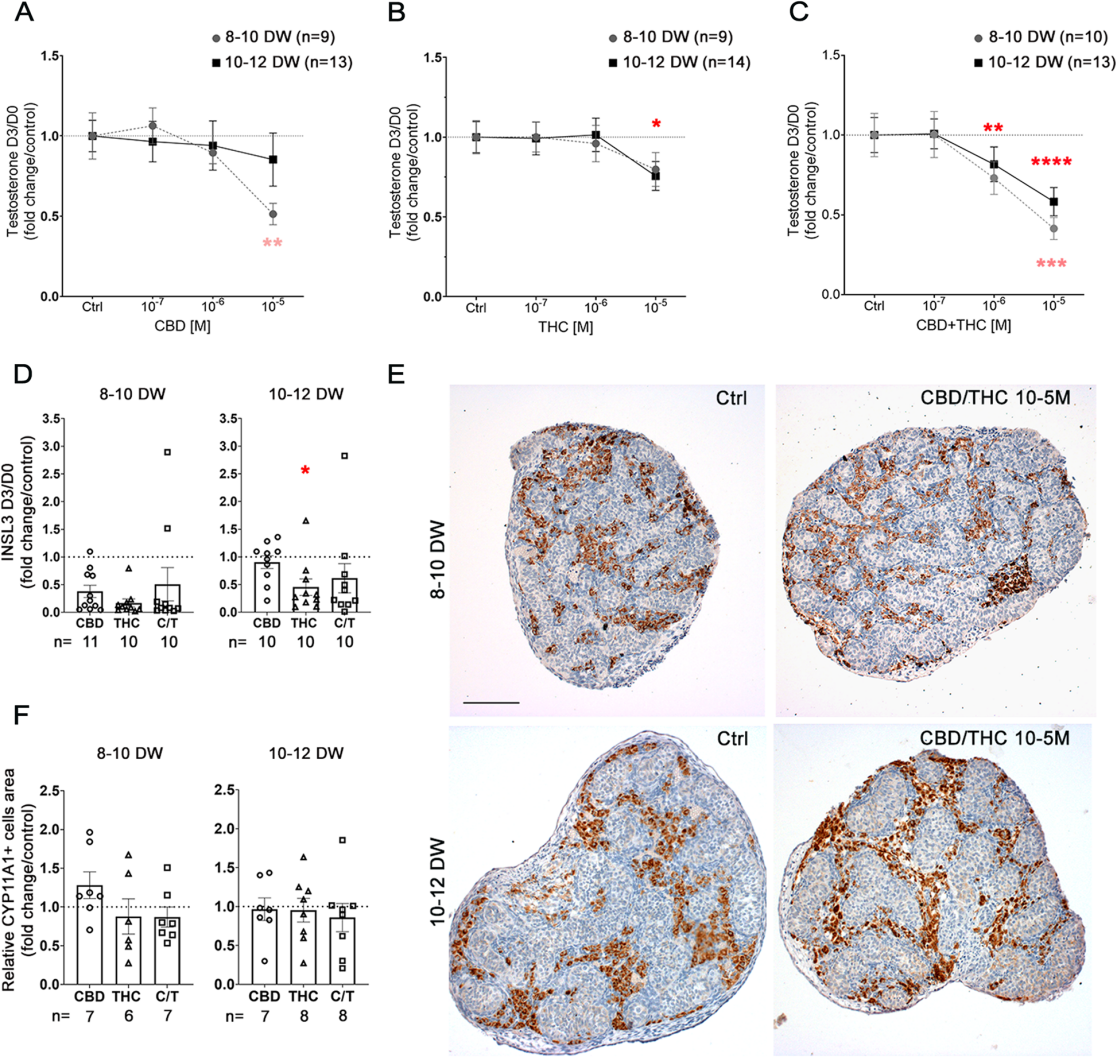
Effect of CBD and/or THC on Leydig cell functions. Amount of testosterone in the supernatants of fetal human testis explants of 8–10 (gray line) or 10–12 (black line) DW cultured for 72 h in presence of controls (DMSO and/or EtOH) or 10^−7^ to 10^−5^ M of (**A**) CDB, (**B**) THC, or (**C**) a mixture of CBD/THC (ratio 1:1). Results are expressed related to the amount of testosterone produced during the first 24 h of culture (D0) and as fold from the respective control. Data are means + / − SEM. An ANOVA test was performed followed by Friedman test for pair comparisons (*, *p* < 0.05; ***, *p* < 0.01; **p < 0.001; ****, *p* < 0.0001). (**D**) Concentration of insulin-like factor 3 (INSL3) in the supernatants of fetal human testis explants of 8–10 or 10–12 DW cultured for 72 h in presence of controls or 10^−5^ M of CBD, THC, or a mixture of CBD/THC (ratio 1:1). Results are expressed related to the amount of INSL3 produced during the first 24 h of culture (D0) and as fold from the respective controls. Data are means + / − SEM. Control and treated conditions were compared two by two using Wilcoxon tests. (*, *p* < 0.05). (**E**) Representative immunostaining of CYP11A1 in 8–10 and a 10–12 DW human fetal testis in controls (DMSO/EtOH) and CBD/THC (ratio 1:1) conditions. CYP11A + cells appear dark brown (DAB staining) in extracordonal tissue, and sections were counterstained with hematoxylin (in blue). No change in terms of Leydig cell morphology was observed. Scale bar = 100 µm. (**F**) Quantification of the surface area occupied by CYP11A1 + Leydig cells related to the section surface after 10^−5^ M of CBD and/or THC treatment compared to respective controls in 8–10 and 10–12 DW embryonic testis

We assessed the impact of 3 days of exposure to various concentrations phytocannabinoids on Leydig cells in 8–10 and 10–12 DW testes. 10^−5^ M of CBD significantly decreased testosterone secretion by testes of 8–10 DW (*p* = 0.0427) (Fig. 5A), while 10^−5^ M of THC significantly decreased testosterone secretion by 10–12 DW testis (*p* = 0.0421) (Fig. 5B). Interestingly, treatment with a mixture of CBD and THC (ratio 1:1) induced a dose-dependent testosterone decrease (Fig. 5C). The pairwise comparisons versus the control condition evidenced a significant decrease starting at 10^−6^ M in the 10–12 DW testes, with an even more pronounced impact at 10^−5^ M (*p* = 0.0005), as well as a highly significant decrease at 10^−5^ M in 8*–*10 DW testes (*p* = 0.0004) for the CBD/THC mix (Fig. 5C). The level of secreted insulin-like factor 3 (INSL3), another Leydig cell-derived hormone, was also significantly decreased in testis explants exposed to 10^−5^ M of THC in 8–10 DW testis, while the effects of THC and CBD/THC did not reach significance in 8–10 or 10–12 DW testes (Fig. 5D). To investigate whether the effect on testosterone secretion was due to a modification of the Leydig cell population, immunohistochemistry was performed using Leydig cell marker CYP11A1. The relative CYP11A1-positive cells area was similar in both 8–10 and 10–12 DW explants treated or not with 10^−5^ M CBD/THC, and no specific change in terms of Leydig cell morphology was noted. In line, the quantification of the relative area occupied by CYP11A1-positive cells did not reveal any significant modifications in 8–10 and 10–12 DW testis treated with 10^−5^ M of either CBD, THC, or the mix (Fig. 5E,F).

### 72 h exposure of human fetal testis to phytocannabinoids ex vivo decreases AMH secretion by Sertoli cells

**Fig. 6:**
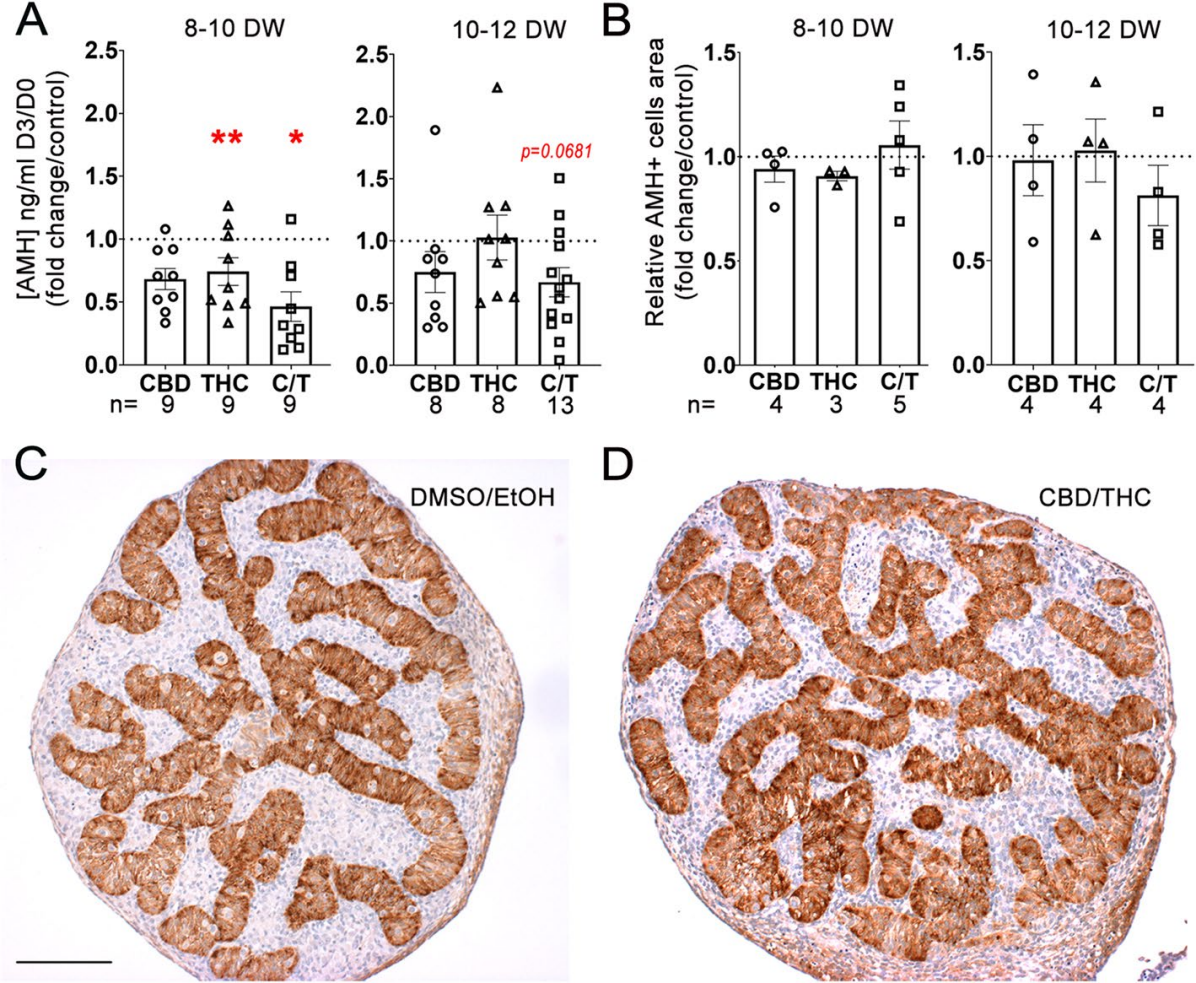
Effect of CBD and/or THC on Sertoli cells functions. (**A**) Amount of AMH in the supernatants of fetal human testis explants of 8–10 or 10–12 DW cultured for 72 h in presence of solvent controls (DMSO and/or EtOH) or 10^−5^ M of CBD and/or THC. Results are expressed related to the amount of AMH produced during the first day of the culture (D0) and as fold from the respective control. Data are means + / − SEM. Control and treated conditions were compared two by two using Wilcoxon tests (*, *p* < 0.05; **, *p* < 0.01). (**B**) Quantification of the surface area occupied by AMH-positive cells (AMH +) after CBD and/or THC treatment at 10^−5^ M compared to respective control in 8–10 and 10–12 DW embryonic testis. Representative immunostaining of AMH, a specific marker of Sertoli cell, in explants of an 8–10 DW human fetal testis treated with (**C**) DMSO/EtOH (control) or (**D**) CBD/THC 10^−5^ M. AMH appears dark brown (DAB staining) in intracordonal tissue, and sections were counterstained with hematoxylin (in blue). Scale bar = 100 µm

The impact of phytocannabinoids on Sertoli cells was first assessed through the analysis of hormonal secretion. AMH secretion by testis explants was significantly decreased in 8–10 DW explants upon treatment with 10^−5^ M of THC (*p* = 0.0078) or CBD/THC (*p* = 0.0195) but was not affected in CBD-treated explants. In 10–12 DW testis cultures, only CBD/THC treatment induced a borderline, non-significant decrease in AMH secretion (*p* = 0.0681) (Fig. 6A). The quantification of the relative surface area occupied by AMH-positive Sertoli cells using immunohistochemistry did not show any modification in testis explants exposed to CBD and/or THC at 10^−5^ M when compared with controls (Fig. 6B) and no apparent change of the distribution of this Sertoli cell marker was observed after CBD/THC treatment at 10^−5^ M (Fig. 6C,D), suggesting that Sertoli cell function rather than viability was affected.

### Several signaling pathways, including those involved in steroid biosynthesis, are modified in the 8–12 DW testes exposed to CBD and/or THC treatment for 72 h

**Fig. 7:**
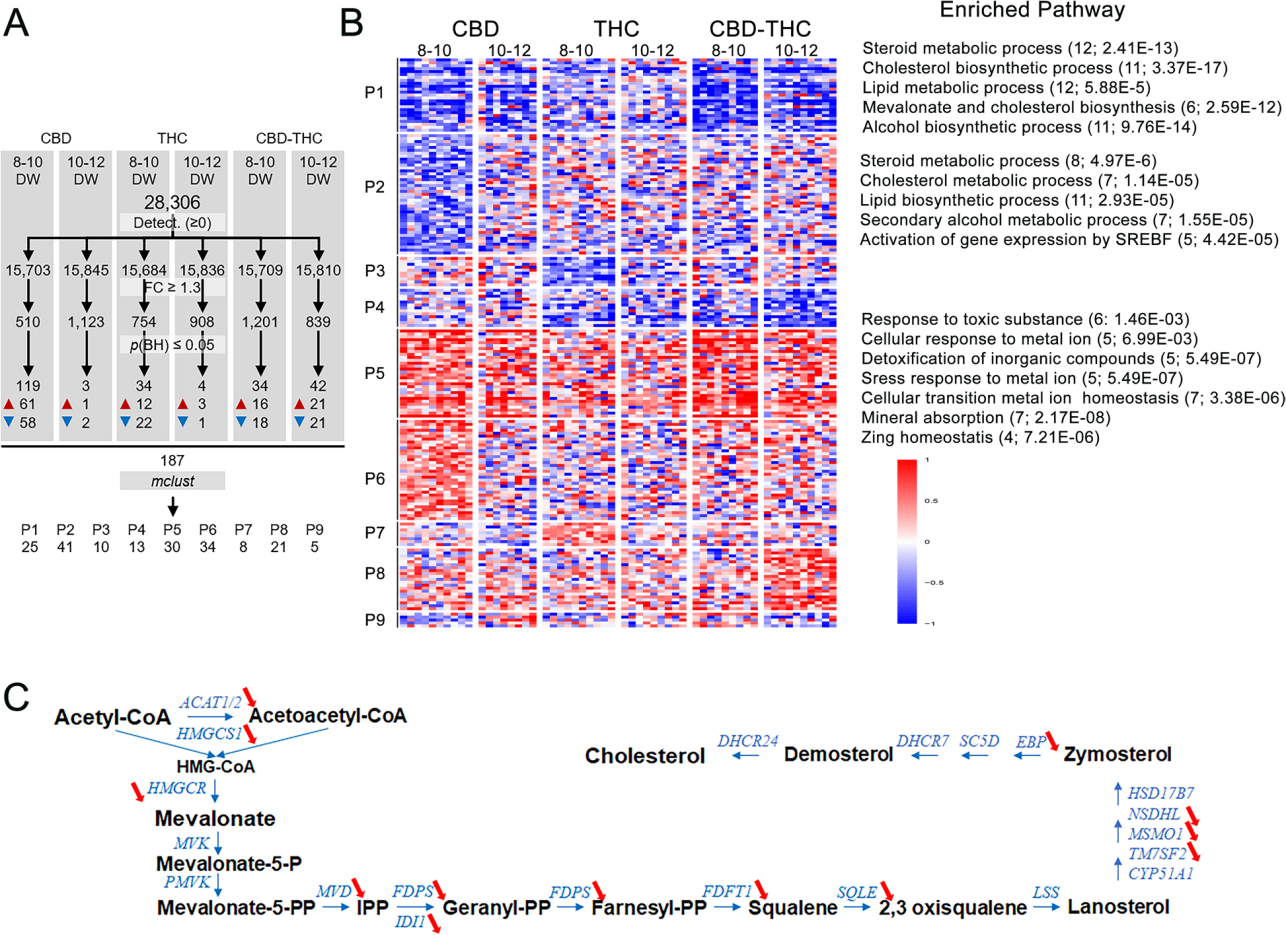
Effect of CBD and THC alone or in mixture on the transcriptome of human fetal testis. (**A**) Analysis of differentially expressed genes (DEG) in 8–10 and 10–12 DW embryonic testis after 72 h exposure to CBD, THC, or CBD/THC at 10^−5^ M, compared to controls. *n* = 10 for each condition. Data statistical analysis shows 187 DEGs in total, either upregulated (red triangle) or downregulated (blue triangle). These DEGs are distributed in 9 patterns (P) of expression, from P1 to P9. (**B**) Heatmap representation of transcriptional changes in 8–10 and 10–12 DW embryonic human testis after 72 h exposure to CBD, THC, or CBD/THC at 10^−5^ M, compared to controls. Heatmap represents the 9 patterns of gene either depleted (down regulated in blue) or enriched (up regulated in red). Genes belonging to enriched pathway are found in P1, P2, and P5 indicated on the right column of the panel. The number of genes belonging to the pathways and *p*-value for each pathway is indicated in bracket. (**C**) Schematic representation of the cholesterol biosynthesis pathway, with genes encoding enzymes indicated in blue. Red arrows indicate the significant decrease of gene expression in 8–10 or 10–12 DW embryonic testis after CBD and/or THC treatment compared to control condition. All of them are deregulated after CBD treatment of 10–12 DW testis

To better understand the effects induced by phytocannabinoids in the fetal testis, we performed 3’ BRB-seq experiments on RNA extracted from 8–10 and 10–12 DW testis explants cultured with either CBD and/or THC at 10^−5^ M for 72 h. Following library preparation and sequencing, and the preprocessing and the normalization of the data, over 15,703 genes were detected in all conditions (Fig. 7A). After comparing each condition with their own control, genes with a fold change ≥ 1.3 and a *p*-value ≤ 0.05 were selected. Upon CBD exposure, 61 genes were up- and 58 downregulated in 8–10 DW testis compared with controls, against only respectively 1 and 2 genes in 10–12 DW testis. Upon THC exposure, 12 genes were up- and 22 downregulated in 8–10 DW testis compared with controls, against respectively 3 and 1 genes in 10–12 DW testis. Upon CBD/THC exposure, 16 genes were up- and 18 downregulated in 8–10 DW testis compared with controls and 21 genes were up- and 21 downregulated in 10–12 DW testis. In total, the expression of 187 genes was modified by the treatments (Additional Table 1). In 8–10 DW, 68.4 and 10.5% of upregulated genes were due to CBD or THC exposure, respectively, and only 5.3% of differential expressed genes (DEGs) were common to the three treatments (CBD, THC or CBD/THC). For downregulated genes, 51.2 and 19.5% of DEG were due to CBD or THC exposure, respectively, and only 3.7% of DEG were common to the three treatments. In 10–12 DW testis, 82.6 and 87% of the up- and downregulated genes were observed in CBD/THC conditions (Additional Fig. 1).

The resulting lists of DEGs were then clustered into distinct gene expression patterns with the mclust algorithm [39]. The analysis resulted in the identification of nine clusters from P1 (25 genes) to P9 (5 genes) (Fig. 7A, B). A Gene Ontology term enrichment analysis was performed with the Annotation Mapping Expression and Network (AMEN) [40] and a list of significantly enriched terms was identified for patterns P1, P2, and P5 (Additional Table 2). The clusters P1 to P4 corresponded mainly to downregulated genes (as *ACAT2; HMGCS1; HMGCR; FDFT1; MSMO1; NSDHL; EBP; SQLE; SC5D; HADH; CYP17A*) involved in steroid biosynthesis pathway, as well as in mevalonate and cholesterol biosynthesis, both necessary to the biosynthesis of steroids (Fig. 7B, C). The clusters from P5 to P9 correspond mainly to upregulated genes (such as *DDIT3; EGR1; NAA60; FACD2; DCLRE1C; RRAGC; STK17B; VEGFB*) involved in the cell responses to toxic substances and inorganic compounds in the human fetal testis (Fig. 7B).

### The deleterious effects of phytocannabinoids are amplified upon a 14-day-long exposure of human fetal testis ex vivo

**Fig. 8:**
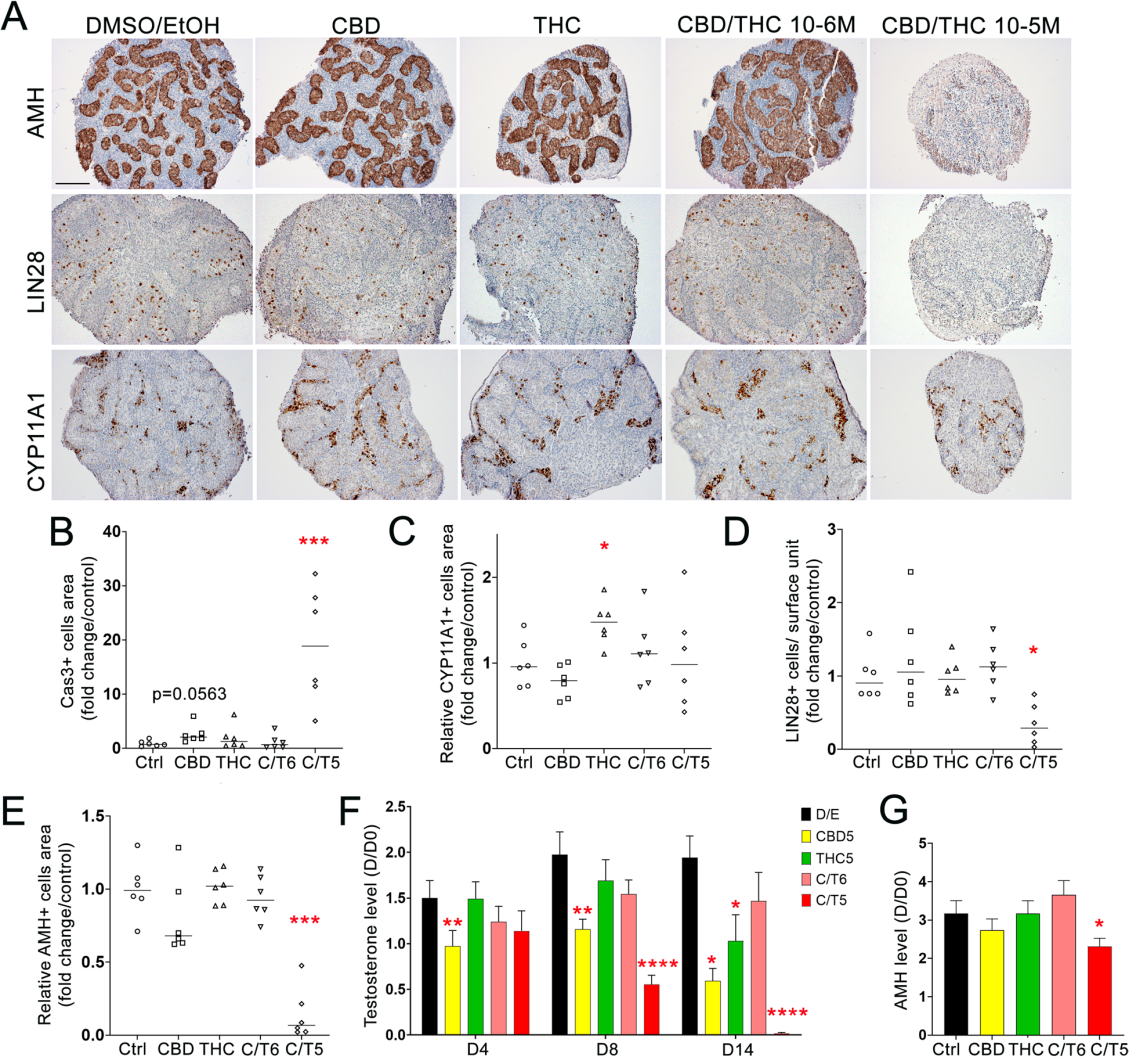
Long-term effect of CBD and/or THC on Leydig cell biology. (**A**) Representative immunostaining of AMH, LIN28A, and CYP11A1 in 10–12 DW testis in dark brown. Sections were counterstained with hematoxylin (in blue). Scale bar = 100 µm. Quantification of the surface area occupied by (**B**) cleaved caspase-3-positive cells (Cas3 +) or (**C**) CYP11A1 + cells after Control (Ctrl), CBD, or THC treatment at 10^−5^ M (CBD, THC) or a mixture of CBD and THC at 10^−5^ M (C/T5) or 10^−6^ M (C/T6) compared to respective control. (**D**) Density of LIN28-positive cells (LIN28 +), resulting from the quantification of positive cells per surface of intracordonal tissue after CBD and/or THC treatment. (**E**) Quantification of the surface area occupied by AMH-positive cells (AMH +) compared to section area after CBD and/or THC treatment. (**F**) Amount of testosterone in the supernatants of 10–12 DW fetal human testis explants cultured for 4 (D4), 8 (D8), and 14 (D14) days or (**G)** for 8 days for AMH in presence of solvent control (DMSO and EtOH) or 10^−5^ M of CDB, THC, or a mixture of CBD/THC (ratio 1:1) at the concentrations of 10^−6^ and 10^−5^ M. Results are expressed related to the amount of testosterone or AMH produced during the first day of the culture (D0). Data are means + / − SEM. An ANOVA test was performed followed by a Freidman test for pair comparisons (*, *p* < 0.05; **, *p* < 0.01;***, *p* < 0.001; ****, *p* < 0.0001)

In an attempt to mimic a regular/chronic use of cannabis during development and try to identify further damages on human embryonic testis, cultures of fetal testes were performed for 2 weeks. Due to the limited amount of human fetal testis tissue, we focused this experiment on 10–12 DW embryonic testis treated or not with either THC 10^−5^ M, CBD 10^−5^ M, CBD/THC 10^−6^ M, and CBD/THC 10^−5^ M during 14 days. Control conditions demonstrate that the overall testis morphology is maintained during the long culture, as shown by normal histology, pattern of expression of Sertoli, germ cells, and Leydig cell markers in situ and absence of signs of abnormal necrosis or apoptosis (Fig. 8A). The 2-week exposure to CBD/THC at 10^−5^ M visually shrank the size of the explants. The measurement of the mean area per explant section confirmed the decrease after exposure to CBD/THC at 10^−5^ M (*p* = 0.0005), but also more modestly by CBD and THC alone at 10^−5^ M (*p* = 0.017 and *p* = 0.068, respectively, Additional Fig. 2). A massive increase in the density of cleaved caspase 3-positive apoptotic cells was obvious in fetal testis explants exposed to CBD/THC at 10^−5^ M, while the increase cell apoptosis induced by CBD at 10^−5^ M was borderline significant (Fig. 8B). The overall cellularity was impacted following exposure to CBD/THC at 10^−5^ M, with an almost disappearance of the cords and a drastic decrease of LIN28 + germ cells and AMH + Sertoli cells, whereas interstitial CYP11A1-positive tissue was preserved (Fig. 8A, C–E). In contrast, CBD and THC alone did not alter the explant cellularity (Fig. 8A) but surprisingly THC induced an enrichment of areas containing CYP11A1-positive Leydig cells, without decreasing the cord surface relative area (Fig. 8C).

The impact of prolonged phytocannabinoid treatment on endocrine function of the testis was investigated at 4 (D4), 8 (D8), and 14 (D14) days after treatment initiation. CBD at 10^−5^ M significantly decreased testosterone levels at all time points, with a more pronounced effect at D14, whereas THC at 10^−5^ M only significantly affected the secretion of testosterone at D14 (Fig. 8F). The mixture of CBD and THC induced a time- and dose-dependent decrease of testosterone, which reached significance at D8 and was completely abolished by D14 for the 10^−5^ M concentration, whereas the 10^−6^ M concentration had no significant effect at any time point (Fig. 8F). At D8, CBD/THC mixture at 10^−5^ M also induced a decrease of the secretion of AMH (Fig. 8G).

## Discussion

This study is the first to demonstrate the presence of the ECS components in the human fetal testis and to reveal an impact of phytocannabinoids on the fetal testis physiology ex vivo. The dynamic regulation of the expression of specific endocannabinoid receptors, endogenous ligands and their respective synthesizing and degrading enzymes during the first months of testis development suggests that ECS signaling plays a role in this process.

HPLC dosages evidenced the presence of the two best-described endogenous ligands of the ECS in 7–12 DW fetal testis, with a 1000 fold higher levels of 2-AG compared with AEA. Similar elevated levels of 2-AG *versus* AEA (up to 1000 times) have been described in the mouse brain [41]. The respective roles of 2-AG and AEA in the human fetal testis are currently unknown. However, the especially high level of 2-AG in the fetal testis suggests a fundamental role in its development. 2-AG being an important metabolite in prostaglandin synthesizing pathway [42], it could also have an ECS-independent mode of action (e.g., inflammation regulation). Both 2-AG and AEA intratesticular levels increased between 7 and 12 DW, albeit only significantly for AEA, likely because of the high inter-individual variability for 2-AG levels. 2-AG in situ detection by MALDI imaging in 11 DW human fetal testis demonstrated its localization primarily within CYP11A1-positive Leydig cell-enriched areas in the interstitial tissue, which contrasts with its localization within the seminiferous tubules of the adult human testis [43]. Similarly to adult testis, we failed to detect AEA in fetal testes in situ, which is probably due to its very low expression level [43].

In agreement with the detection of 2-AG and to a lower level of AEA, our analysis of human fetal testis bulk RNA-seq data demonstrated that 6–17 DW testes express the synthesizing and degrading enzymes of these molecules in variable quantities. 2-AG synthesis is first mediated by PLC enzymes [44, 45], which isoforms *PLCG1* and *PLCD1* were detected between 7 and 12 DW. Likewise, we demonstrated the expression of 2-AG main synthesizing (*DAGLA* and *B*) and degrading enzymes (*MAGL*). Levels of 2-AG increased between 7 and 12 DW but did not reach significance, consistent with the stable expression of *PLCG1, PLCD1, DAGLA, DAGLB,* and *MAGL* enzymes. The expression of *ABDH12* [46], another 2-AG degrading enzyme, was also stable from 9 to 12 DW. ABHD2 is an alternative degrading enzyme of 2-AG [47], expressed by adult testicular germ cell, in which it may regulate meiosis entry [43]. The increase of its expression between 7 and 12 DW suggest that this enzyme is not primarily involved in 2-AG degradation in fetal testis at this time. AEA main synthesizing enzymes *NAT10* and *NAPE-PLD* were also present in the fetal testis, as well as its main degrading enzyme, *FAAH*. However, the decreased expression of *NAT10* concomitantly to the increased expression of *FAAH* between 6 and 12 DW, a time window during which AEA levels significantly increased, suggest that AEA synthesis and degradation occurs through alternative pathways in the first trimester testes. As a matter of fact, we detected the stable expression of two AEA alternative synthesizing enzymes (*ABHD4* and *GDE1*) [48, 49] and two AEA alternative degrading enzymes (*FAAH2* and *CYPXN1*) [50–52] in testes from 6 to 12 DW.

The analysis of RNA-seq data additionally highlighted the expression in the human fetal testis of the genes *CNR1* and *CNR2*, which encode the two main cannabinoid receptors, CB1 and CB2. *CNR1* expression was variable between 7 and 17 DW and overall higher than *CNR2,* as confirmed by RT-qPCR. In 10 to 12 DW testis, in situ hybridization using RNAscope, a recognized highly specific and sensitive technique, demonstrated the expression of *CNR1* mainly in gonocytes and Leydig cells, and to a lower level in Sertoli cells. Small dots (each dot represent the detection of an RNA copy) corresponding to *CNR2* were occasionally observed in these three cell types. Biggest dots were observed in the case of *CNR1* meaning the amount of *CNR1* RNA is more important. CB1 and CB2 detection in the fetal testis by immunohistochemisty was not successful, which could be due to their relatively low expression levels combined with the high background staining generated by the available antibodies. Similarly, in the adult testis, immunohistochemistry of CB2 generated high background staining and only post-meiotic germ cells appeared strongly labelled [43]. In our study, the localization of *CNR1* in Leydig cells and gonocytes indicate both these cell types could be sensitive to 2-AG, a highly effective agonist of CB1 [53] mainly synthesized by fetal Leydig cells. This uncovers a potential new autocrine and paracrine signaling pathways between these two cell types. In addition, we detected several cannabinoid alternative receptors by RNA-seq analysis, which could also be involved in this signaling pathway (data not shown).

In pregnant women, THC, CBD, and their metabolites quickly cross the placenta barrier and are found in the umbilical cord [5], cord blood, or infant urine and hair after delivery [54]. The use of cannabis during pregnancy and breastfeeding can induce negative birth outcomes such as an increased risk of prematurity, a reduced birth weight and cognitive deficits in children [7, 55, 56]. To date, its effect on the genital tract and on the secretion of reproductive hormones during development, required for the differentiation of male genital organs and ducts and for brain masculinization, has never been investigated [8]. Despite the deleterious outcomes reported in the fetus, there are currently no data available regarding the exact concentrations of cannabis compounds that the human fetus may be exposed to. Moreover, the peak of plasma concentrations (Cmax) differs depending on the mode of administration of cannabis and the content of THC. THC Cmax after smoking a cannabis cigarette is around 10^−7^ M to 7.4 × 10^−7^ M for cigarettes containing between 19 and 69.4 mg of THC (Ohlsson et al. 1980; Kauert et al. 2007; Hunault et al. 2008), while CBD Cmax after smoking a cannabis cigarette are higher, ranging from 1.8 × 10^−5^ M to 2.9 × 10^−5^ M for 1.5 mg CBD ingested [57]. The placenta transfer of cannabinoid components is considered to be very high [58]. Thus studies performed on animal models showed that the concentration of THC can be equal between fetal and maternal plasma 3 h after THC administration and that CBD transfer to the fetus began 15 min after CBD administration with a rate of 66.9% [11, 59]. Based on these data, we speculate that the concentrations of 10^−7^ to 10^−5^ M used in this study could reflect the range of concentrations of cannabis compounds that the fetus may be exposed to upon maternal consumption, with 10^−5^ M at the high end.

Using our original and well characterized ex vivo model of human fetal testis [24, 25, 28, 29], we evaluated the effects of the main components of cannabis, CBD, and THC on 8–12 DW testes. This model has already enabled us to identify the toxic effects of environmental compounds visible at the macroscopic level in short-term culture [25, 60]. Here, we provide the first evidence that 72 h exposure of testis explants to phytocannabinoids induce a range of deleterious effect. While the morphology of the tissue was not affected by the treatment, even at the highest concentrations, and no massive toxicity was observed, the number of apoptotic cells in intracordonal compartments increased, fetal testicular cell proliferation decreased, and both testosterone secretion by Leydig cells and AMH secretion by Sertoli cells diminished. BRB-seq transcriptomic analysis of phytocannabinoid-exposed testis explants revealed 187 differentially expressed genes (DEGs) upon treatment, of which key genes for steroid synthesis and toxic substance response. The observed effects varied depending on the molecules (CBD or THC) and age of the tissue, probably in relation with the complex interactions between ligands and cannabinoid receptors [14]. THC is described as a partial agonist with high affinity for both CB1 and CB2, while CBD is thought to be a negative allosteric modulator for CB1. CBD displays a weak inverse agonism for CB2 and may also act as an “indirect” CB1/CB2 agonist by increasing the level of 2-AG or by weakly inhibiting AEA enzymatic hydrolysis. CB1 and CB2 are both coupled to G proteins and activate a number of intracellular signaling pathways [14].

As showed by our data, alteration of testosterone secretion was not due to a modification of Leydig cell density after 72 h, as it remained stable. Moreover, CBD alone did not induce a significant decrease of proliferating cell density in extracordonal tissue of 8–10 DW testes, nor did THC in 10–12 DW testes, although these compounds affected testosterone levels at these respective ages. On the one hand, the accentuation of the decrease in testosterone production as a function of the concentration at 3 days of exposure, and over time in the presence of CBD, without modification of the relative area of the CYP11A1-positive Leydig cells suggests that the steroidogenic function more than the nature and/or differentiation of these cells is altered by CBD. This is consistent with transcriptomic data that indicate an alteration of central lipid metabolism in CBD-exposed cells. The absence of major alteration of the Sertoli endocrine function suggests a specific action of CBD on lipid metabolism. The anti-androgenic effects of THC on the production of testosterone are more tenuous, depend on age, and are only visible in the 10–12 DW-exposed testes after 2 weeks of exposure. The fact that it is accompanied by a relative increase in the surface occupied by CYP11A1-positive Leydig cells without the surface of the cords being altered suggests that the proportion of Leydig cells within the interstitial tissue is modified. It is possible that Leydig cells proliferate or differentiate in compensation for the decrease in their ability to synthesize testosterone. These data suggest that the individual effect of CBD and THC on testosterone secretion occurred at the molecular level. Nevertheless, the increased effect of the THC/CBD mix on testosterone might be due to a combination of both molecular and cellular effect, since a decreased proliferation was observed upon exposure to CBD/THC in 8–10 and 10–12 DW testes. The alteration in testicular cell viability visible from 3 days of exposure to the mix at 10^−5^ M culminates in an overall shrinkage of the tissue at 14 days. Total cord depletion after 14 days of exposure, targeting both Sertoli and germ cells, materializes from 3 days of exposure onwards with an increase in intracordonal apoptosis. The concentration-dependent decrease in endocrine function of testosterone at 3 days is concomitant to a decrease in the proliferation of interstitial cells. This suggests that the effects of CBD on cell metabolism, and of THC on the cell differentiation/function balance are additive to the detriment of tissue viability. In line with the hypothesis of a specific impact of cannabinoids on testosterone production by the fetal testis, anti-androgenic effect of CBD and THC alone have been demonstrated in embryonic rodent in vivo [61–63]. Here we reveal that direct exposure of human fetal testis to CBD and/or THC is harmful to testosterone secretion, likely as a result of the alteration of steroid biosynthesis by Leydig cells. This hypothesis is supported by our transcriptomic analysis of testis explants after phytocannabinoid treatments, which evidenced a downregulated expression of numerous genes involved in steroids and cholesterol synthesis after a CBD/THC exposure, as it has been previously shown after phthalate exposure of human fetal testis [64]. For instance, the expression of cytochrome P450 family 17 subfamily A member 1 (CYP17A1), a key testosterone biosynthesis enzyme, was significantly decreased after exposure of 10–12 DW testicular explants to CBD/THC mixture, while expression of enzymes like *HSD3B1/2* or *CYP11A1* were not altered. Our results are coherent with other studies that show the effect of cannabinoid components on steroid synthesis. In the rat, in vitro experiments showed that cannabinoids affect the secretion of steroids such as testosterone [65–67], progesterone, or estradiol [67]. In vivo, THC administration given during the third week of gestation in rat significantly blocked the surge of testosterone occurring in male fetus [68].

In parallel, the expression of genes involved in the response to toxic substance, detoxification and cellular stress significantly increased. Cell metabolism alterations might be responsible for cell toxicity in longer-term culture, as suggested by the massive apoptosis observed in fetal testis exposed to CBD/THC for 14 days. As the majority of effects are observed in 10^−5^ M CBD/THC condition, a non-specific toxic effect on testicular cords cannot be excluded. Nevertheless, the presence of ECS in human fetal testis, the absence of macroscopic signs of tissue toxicity, the absence of obvious changes in the number of testis cell types, and the deregulation of steroid synthesis pathway suggest that the observed effects could be due to a specific interaction of phytocannabinoids with the ECS. Of note, the overall absence of gene expression alteration with THC alone exposure could be due to the chosen BRB-seq fold change threshold impairing the detection of more discrete gene expression modification, or, alternatively, to post-translational mechanisms. Surprisingly also, while the mix of THC and CBD at 10^−5^ M for 72 h impacted a similar number of genes in 8–10 DW and 10–12 DW testicular explants, CBD or THC alone led to very few variations of gene expression in the older testes. This might reflect an exacerbated window of sensitivity in the 8–10 DW testis (as observed for CBD specifically impacting testosterone production at this early age for instance), the targeting of distinct cell populations or their under-representation in older testes, and/or differences in cell metabolism between these two developmental ages. The exacerbated effects of the mixture when compared with the individual molecules may be explained by the interaction of cannabis compounds with cannabinoid receptors and the activation through CB1 and CB2 of signaling pathways involved in the regulation of apoptosis and cell proliferation and by an additive effect of CBD and THC [14].

In addition to testosterone decrease, our results demonstrate an effect of phytocannabinoids on AMH secretion in short-term cultures, without any alteration of Sertoli cell density assessed by AMH staining. Of note, cell markers can still be detectable in apoptotic cells [60]. Since the number of apoptotic cells increased in intracordonal tissue, we believe that the cannabinoid treatments affected the microenvironment necessary to the homeostasis of germ cell and Sertoli cell populations. The increased cell death in intracordonal tissue is consistent with the alteration of human adult Sertoli cell viability reported in another study after an in vitro exposure to CBD from 7.10^−6^ M [69]. While THC alone had no negative impact on Sertoli cell activity in another study, the highest dose tested (6.4 0.10^−7^ M) was lower than the 10^−5^ M used here [70]. We investigated the expression of genes involved in AMH synthesizing pathways in testicular explants exposed with CBD and/or THC at 10^−5^ M for 72 h by BRB-seq, but failed to observe any significant modification of gene expression. This may suggest a post-translational effect of cannabinoids on AMH secretion linked to a globally altered Sertoli cell metabolism, since an alteration of genes involved in cell metabolism was observed in BRB-seq experiments on whole testis explants. The alteration of Sertoli cells by CBD/THC mixture was confirmed after 14 days of treatment in our model through the disappearance of this somatic cell type and the significant decrease of AMH secretion. Globally, major deleterious effects were observed on cell viability and growth inside the cords, and on Leydig cell function outside the cords, consistent with the localization of CB1 receptors. The depletion of germ cells in long-term cultures could therefore be due to an indirect effect of the drugs on Sertoli cells and on testosterone production by Leydig cells and/or to a direct effect on this cell population.

The development of TGCT, which partly originates from a dysregulation of fetal germ cell differentiation [71], has been associated with cannabis consumption in men [19–21] but the mechanisms at play are unknown. The elevated incidence of TGCT these last decades is suspected to be linked to exposure of the fetal testis to a range of chemicals through maternal impregnation [72], with subsequent environmental exposures during teenage or adulthood acting in some cases as a secondary trigger [73]. Indeed, the differentiation of germ cells is mainly influenced by the hormonal secretions of Leydig and Sertoli cells, which were affected in our study by cannabis component exposure. Animal models and data in men point at a role of Leydig cell dysfunction and subsequent decreased testosterone level production in the etiology of TGCT [74, 75]. The imbalance of the cellular microenvironment could potentially result in an impaired gonadal development, an arrest of gonocyte differentiation and the formation of germ cell neoplasia in situ (GNIS) that can promote neoplastic transformation later in life [72]. Altogether, these elements suggest that phytocannabinoids might have a deleterious effect on germ cells during fetal life and represent secondary triggers for cancer when used in men after puberty. Further studies, for instance using single-cell RNA seq, are warranted to better apprehend the mechanisms underlying the effects of cannabis component on the genome of rare population of pluripotent germ cells in fetal human testis.

## Conclusions

In conclusion, this study unveils the expression of the ECS in fetal human testis and shows a direct negative impact of CBD and/or THC at 10^−5^ M on several testicular cellular types’ functions and viability ex vivo. Importantly, 8–12 DW is a recognized critical period of testicular development particularly sensitive to environmental exposures [76–78]. Further studies are warranted to explore the in vivo relevance of the cannabinoid concentrations used. Whether the effects of THC and CBD on Leydig cells and Sertoli cells result from CB1 and/or CB2 signaling also require additional studies, e.g., using gene silencing or specific agonists/antagonists of CB1 and CB2 and alternate receptors in isolated testicular cells. Cannabis consumption by pregnant women has risen worldwide over the past decades and legalization for its recreational and therapeutic purposes is debated in numerous countries. An understanding of the potential adverse effects of exposures to cannabis components on the establishment of the male reproductive functions during development is urgently needed. Our study highlights potential deleterious effects of cannabinoids on the developing testis and paves the way for further deciphering of the impact of cannabinoids at the molecular and cellular levels in the fetus. In addition, large-scale mother–child cohorts are required to study the impact of maternal cannabis consumption on the occurrence of reproductive pathologies, from birth to adulthood.

## Supplementary Information

**Additional file 1: Figure 1.** Venn diagrams representation of DEGs after CBD and/or THC treatments.

**Additional file 2: Figure 2.** Mean area of fetal testis explants after culture with CBD and/or THC. Results are expressed related to the mean explant area. Data are means +/- SEM. Control and treated conditions were compared two by two using Wilcoxon tests.

**Additional file 3: Table 1.** List of DEGs after CBD and/or THC treatments.

**Additional file 4: Table 2.** Gene Ontology term enrichment analysis of the DEGs.

## Data Availability

The datasets used and/or analyzed during the current study are available from the corresponding author on reasonable request.

## Abbreviations

ABHD2, 4, and 12: Abhydrolase domain-containing protein 2, 4, and 12
2-AG: 2-Arachidonyl-glycerol
AEA: Anandamide
AMEN: Annotation Mapping Expression and Network
AMH: Anti-Müllerian Hormone
APCI: Atmospheric pressure chemical ionization
ARA: Arachidonic acid
BSA: Bovine serum albumin
CA: Cannabinoids
CB: Cannabinoid receptors
CBD: Cannabidiol
CYP4X1: Cytochrome P450 4X1
DAB: 3,3’-Diaminobenzidine tetrahydrochloride
DAGLA: Diacylglycerol lipase-alpha
DAGLB: Diacylglycerol lipase-beta
D0: First 24 h of culture
DMSO: Dimethylsulfoxide
DEGs: Differential expressed genes
DW: Developmental weeks
eCA: Endocannabinoid
ECS: Endocannabinoid system
ELISA: Enzyme-linked immunosorbent assay
EtOH: Ethanol
FAAH: Fatty acid amide hydrolase
FDR: False discovery rate
FPKM: Fragments Per Kilobase Million
GDE1: Glycerophosphodiester phosphodiesterase 1
GlyEth: Glycerophosphoacylethanolamides
GNIS: Germ cell neoplasia in situ
INSL3: Insulin-like factor 3
ITS: Insulin, transferrin, selenium
MAGL: Monoglyceride lipase
NAPE-PLD: N-acyl-phosphatidylethanolamine-hydrolyzing phospholipase D
NAT10: N-acetyltransferase 10
TCA: Trichloroacetic acid
TGCTs: Testicular germ cell tumors
THC: Δ9-Tetrahydrocannabinol
PC: Phosphatidylcholine
PCA: Principal component analysis
PEA: Palmitoyethanolamide
PGCs: Primordial germ cells
PLCG1: Phospholipase C-gamma-1
PLCD1: Phospholipase C-delta-1
PBS: Phosphate buffer saline
RT: Room temperature
UMI: Unique molecular identifier
UMAP: Uniform *Manifold* Approximation and Projection

## References

[1] Young-Wolff KC, Sarovar V, Tucker LY, Avalos LA, Alexeeff S, Conway A (2019). Trends in marijuana use among pregnant women with and without nausea and vomiting in pregnancy, 2009–2016. Drug Alcohol Depend.

[2] Jarlenski M, Spencer N (2022). Perceptions of safety around use of cannabis and nicotine/tobacco in pregnancy. Clin Obstet Gynecol.

[3] Volkow ND, Han B, Compton WM, McCance-Katz EF (2019). Self-reported medical and nonmedical cannabis use among pregnant women in the United States. JAMA.

[4] Lozano J, García-Algar O, Marchei E, Vall O, Monleon T, Giovannandrea RD (2007). Prevalence of gestational exposure to cannabis in a Mediterranean city by meconium analysis. Acta Paediatrica.

[5] Kim J, de Castro A, Lendoiro E, Cruz-Landeira A, López-Rivadulla M, Concheiro M (2018). Detection of in utero cannabis exposure by umbilical cord analysis. Drug Test Anal.

[6] Marchand G, Masoud AT, Govindan M, Ware K, King A, Ruther S (2022). Birth outcomes of neonates exposed to marijuana in utero: a systematic review and meta-analysis. JAMA Netw Open.

[7] Day NL, Richardson GA, Goldschmidt L, Robles N, Taylor PM, Stoffer DS (1994). Effect of prenatal marijuana exposure on the cognitive development of offspring at age three. Neurotoxicology and Teratology mars.

[8] Lo JO, Hedges JC, Girardi G (2022). Impact of cannabinoids on pregnancy, reproductive health, and offspring outcomes. Am J Obstet Gynecol.

[9] Jaques SC, Kingsbury A, Henshcke P, Chomchai C, Clews S, Falconer J (2014). Cannabis, the pregnant woman and her child: weeding out the myths. J Perinatol.

[10] Grotenhermen F (2003). Pharmacokinetics and pharmacodynamics of cannabinoids. Clin Pharmacokinet.

[11] Hutchings DE, Martin BR, Gamagaris Z, Miller N, Fico T (1989). Plasma concentrations of delta-9-tetrahydrocannabinol in dams and fetuses following acute or multiple prenatal dosing in rats. Life Sci.

[12] Harbison RD, Mantilla-Plata B (1972). Prenatal toxicity, maternal distribution and placental transfer of tetrahydrocannabinol. J Pharmacol Exp Ther.

[13] Monfort A, Ferreira E, Leclair G, Lodygensky GA (2022). Pharmacokinetics of cannabis and its derivatives in animals and humans during pregnancy and breastfeeding. Front Pharmacol.

[14] Ligresti A, De Petrocellis L, Di Marzo V (2016). From phytocannabinoids to cannabinoid receptors and endocannabinoids: pleiotropic physiological and pathological roles through complex pharmacology. Physiol Rev.

[15] Grimaldi P, Di Giacomo D, Geremia R. The endocannabinoid system and spermatogenesis. Front Endocrinol. 2013;4. Disponible sur: http://journal.frontiersin.org/article/10.3389/fendo.2013.00192/abstract. [Cité 27 sept 2022]. 10.3389/fendo.2013.00192 PMC3864102 24379805

[16] Bari M (2011). The manifold actions of endocannabinoids on female and male reproductive events. Front Biosci.

[17] De Domenico E, Todaro F, Rossi G, Dolci S, Geremia R, Rossi P (2017). Overactive type 2 cannabinoid receptor induces meiosis in fetal gonads and impairs ovarian reserve. Cell Death Dis.

[18] Da Silva J, Dochez-Arnault J, Dedoist-Letimonier C, Dejucq-Rainsford N, Gely-Pernot A. The acute exposure of human adult testis tissue to cannabinoids THC and CBD does not impact testosterone production nor germ cell lineage. World J Men’s Health. 2023;41:e34. 10.5534/wjmh.220210 PMC10523127 37118951

[19] Gurney J, Shaw C, Stanley J, Signal V, Sarfati D (2015). Cannabis exposure and risk of testicular cancer: a systematic review and meta-analysis. BMC Cancer.

[20] Callaghan RC, Allebeck P, Akre O, McGlynn KA, Sidorchuk A (2017). Cannabis use and incidence of testicular cancer: a 42-year follow-up of Swedish men between 1970 and 2011. Cancer Epidemiol Biomarkers Prev.

[21] Song A, Myung NK, Bogumil D, Ihenacho U, Burg ML, Cortessis VK (2020). Incident testicular cancer in relation to using marijuana and smoking tobacco: a systematic review and meta-analysis of epidemiologic studies. Urol Oncol.

[22] Fisher JS (2003). Human « testicular dysgenesis syndrome »: a possible model using in-utero exposure of the rat to dibutyl phthalate. Hum Reprod.

[23] Joensen UN, Jørgensen N, Rajpert-De Meyts E, Skakkebaek NE (2008). Testicular dysgenesis syndrome and Leydig cell function. Basic Clin Pharmacol Toxicol.

[24] Mazaud-Guittot S, Nicolaz CN, Desdoits-Lethimonier C, Coiffec I, Maamar MB, Balaguer P (2013). Paracetamol, aspirin, and indomethacin induce endocrine disturbances in the human fetal testis capable of interfering with testicular descent. J Clin Endocrinol Metabol.

[25] Maamar MB, Lesné L, Desdoits-Lethimonier C, Coiffec I, Lassurguère J, Lavoué V (2015). An investigation of the endocrine-disruptive effects of Bisphenol A in human and rat fetal testes. Nadal A, éditeur. PLoS One.

[26] Kauert GF, Ramaekers JG, Schneider E, Moeller MR, Toennes SW (2007). Pharmacokinetic properties of 9-tetrahydrocannabinol in serum and oral fluid. J Anal Toxicol.

[27] Grant KS, Petroff R, Isoherranen N, Stella N, Burbacher TM (2018). Cannabis use during pregnancy: pharmacokinetics and effects on child development. Pharmacol Ther.

[28] Ben Maamar M, Lesné L, Hennig K, Desdoits-Lethimonier C, Kilcoyne KR, Coiffec I (2017). Ibuprofen results in alterations of human fetal testis development. Sci Rep.

[29] Desdoits-Lethimonier C, Albert O, Bizec BL, Perdu E, Zalko D, Courant F (2012). Human testis steroidogenesis is inhibited by phthalates. Hum Reprod.

[30] Lecluze E, Rolland AD, Filis P, Evrard B, Leverrier-Penna S, Maamar MB (2020). Dynamics of the transcriptional landscape during human fetal testis and ovary development. Hum Reprod.

[31] Draskau MK, Lardenois A, Evrard B, Boberg J, Chalmel F, Svingen T (2021). Transcriptome analysis of fetal rat testis following intrauterine exposure to the azole fungicides triticonazole and flusilazole reveals subtle changes despite adverse endocrine effects. Chemosphere.

[32] Giacosa S, Pillet C, Séraudie I, Guyon L, Wallez Y, Roelants C (2021). Cooperative blockade of CK2 and ATM kinases drives apoptosis in VHL-deficient renal carcinoma cells through ROS overproduction. Cancers.

[33] Alpern D, Gardeux V, Russeil J, Mangeat B, Meireles-Filho ACA, Breysse R (2019). BRB-seq: ultra-affordable high-throughput transcriptomics enabled by bulk RNA barcoding and sequencing. Genome Biol.

[34] Love MI, Huber W, Anders S (2014). Moderated estimation of fold change and dispersion for RNA-seq data with DESeq2. Genome Biol déc.

[35] Edgar R (2002). Gene Expression Omnibus: NCBI gene expression and hybridization array data repository. Nucleic Acids Research.

[36] Lê S, Josse J, Husson F. FactoMineR : an *R* package for multivariate analysis. J Stat Soft. 2008;25(1). Disponible sur: http://www.jstatsoft.org/v25/i01/. [Cité 27 sept 2022].

[37] Benjamini Y, Hochberg Y (1995). Controlling the false discovery rate: a practical and powerful approach to multiple testing. J R Stat Soc Series B (Methodological).

[38] Darde TA, Gaudriault P, Beranger R, Lancien C, Caillarec-Joly A, Sallou O (2018). TOXsIgN: a cross-species repository for toxicogenomic signatures. Wren J, éditeur. Bioinformatics.

[39] Scrucca L, Fop M, Murphy TB, Raftery AE (2016). mclust 5: clustering, classification and density estimation using Gaussian finite mixture models. R J.

[40] Chalmel F, Primig M (2008). The Annotation, Mapping, Expression and Network (AMEN) suite of tools for molecular systems biology. BMC Bioinformatics.

[41] Schurman LD, Lu D, Kendall DA, Howlett AC, Lichtman AH. Molecular mechanism and cannabinoid pharmacology. In: Nader MA, Hurd YL, éditeurs. Substance Use Disorders. Cham: Springer International Publishing. Handb Experimental Pharmacology 2019;258:323‑53. Disponible sur: http://link.springer.com/10.1007/164_2019_298. [Cité 26 juill 2022]. 10.1007/164_2019_298 PMC8637936 32236882

[42] Nomura DK, Morrison BE, Blankman JL, Long JZ, Kinsey SG, Marcondes MCG (2011). Endocannabinoid hydrolysis generates brain prostaglandins that promote neuroinflammation. Science.

[43] Nielsen JE, Rolland AD, Rajpert-De Meyts E, Janfelt C, Jørgensen A, Winge SB (2019). Characterisation and localisation of the endocannabinoid system components in the adult human testis. Sci Rep.

[44] Ueda N, Tsuboi K, Uyama T, Ohnishi T (2011). Biosynthesis and degradation of the endocannabinoid 2-arachidonoylglycerol. BioFactors janv.

[45] Murataeva N, Straiker A, Mackie K (2014). Parsing the players: 2-arachidonoylglycerol synthesis and degradation in the CNS: 2-AG synthesis and degradation in the CNS. Br J Pharmacol mars.

[46] Blankman JL, Simon GM, Cravatt BF (2007). A comprehensive profile of brain enzymes that hydrolyze the endocannabinoid 2-arachidonoylglycerol. Chem Biol.

[47] Miller MR, Mannowetz N, Iavarone AT, Safavi R, Gracheva EO, Smith JF (2016). Unconventional endocannabinoid signaling governs sperm activation via the sex hormone progesterone. Science.

[48] Simon GM, Cravatt BF (2006). Endocannabinoid biosynthesis proceeding through glycerophospho-N-acyl ethanolamine and a role for α/β-hydrolase 4 in this pathway. J Biol Chem.

[49] Simon GM, Cravatt BF (2010). Characterization of mice lacking candidate N-acyl ethanolamine biosynthetic enzymes provides evidence for multiple pathways that contribute to endocannabinoid production in vivo. Mol BioSyst.

[50] Snider NT, Walker VJ, Hollenberg PF (2010). Oxidation of the endogenous cannabinoid arachidonoyl ethanolamide by the cytochrome P450 monooxygenases: physiological and pharmacological implications. Pharmacol Rev.

[51] Urquhart P, Nicolaou A, Woodward DF (2015). Endocannabinoids and their oxygenation by cyclo-oxygenases, lipoxygenases and other oxygenases. Biochim et Biophys Acta.

[52] Wei BQ, Mikkelsen TS, McKinney MK, Lander ES, Cravatt BF (2006). A second fatty acid amide hydrolase with variable distribution among placental mammals. J Biol Chem.

[53] Hillard CJ (2000). Biochemistry and pharmacology of the endocannabinoids arachidonylethanolamide and 2-arachidonylglycerol. Prostaglandins Other Lipid Mediat.

[54] Ostrea EM, Knapp DK, Tannenbaum L, Ostrea AR, Romero A, Salari V (2001). Estimates of illicit drug use during pregnancy by maternal interview, hair analysis, and meconium analysis. J Pediatr.

[55] Hayakawa K, Mishima K, Hazekawa M, Sano K, Irie K, Orito K (2008). Cannabidiol potentiates pharmacological effects of Δ9-tetrahydrocannabinol via CB1 receptor-dependent mechanism. Brain Res.

[56] Conner SN, Bedell V, Lipsey K, Macones GA, Cahill AG, Tuuli MG (2016). Maternal marijuana use and adverse neonatal outcomes: a systematic review and meta-analysis. Obstet Gynecol.

[57] Swortwood MJ, Newmeyer MN, Andersson M, Abulseoud OA, Scheidweiler KB, Huestis MA (2017). Cannabinoid disposition in oral fluid after controlled smoked, vaporized, and oral cannabis administration: oral fluid cannabinoids after three routes of cannabis administration. Drug Test Anal.

[58] Little BB, VanBeveren TT (1996). Placental transfer of selected substances of abuse. Sem Perinatol.

[59] Ochiai W, Kitaoka S, Kawamura T, Hatogai J, Harada S, Iizuka M (2021). Maternal and fetal pharmacokinetic analysis of cannabidiol during pregnancy in mice. Drug Metab Dispos.

[60] Gaudriault P, Mazaud-Guittot S, Lavoué V, Coiffec I, Lesné L, Dejucq-Rainsford N (2017). Endocrine disruption in human fetal testis explants by individual and combined exposures to selected pharmaceuticals, pesticides, and environmental pollutants. Environ Health Perspect.

[61] Dalterio S, Bartke A (1981). Fetal testosterone in mice: effect of gestational age and cannabinoid exposure. J Endocrinol.

[62] Dalterio SL, deRooij DG (1986). Maternal cannabinoid exposure effects on spermatogenesis in male offspring. Int J Androl août.

[63] Murphy LL, Gher J, Szary A (1995). Effects of prenatal exposure to delta-9-tetrahydrocannabinol on reproductive, endocrine and immune parameters of male and female rat offspring. Endocr déc.

[64] Muczynski V, Lecureuil C, Messiaen S, Guerquin MJ, N’Tumba-Byn T, Moison D (2012). Cellular and molecular effect of MEHP involving LXRα in human fetal testis and ovary. Singh SR, éditeur. PLoS One.

[65] List A, Nazar B, Nyquist S, Harclerode J (1977). The effects of delta9-tetrahydrocannabinol and cannabidiol on the metabolism of gonadal steroids in the rat. Drug Metab Dispos.

[66] Jakubovic A, McGeer EG, McGeer PL (1979). Effects of cannabinoids on testosterone and protein synthesis in rat testis Leydig cells in vitro. Mol Cell Endocrinol.

[67] Reich R, Laufer N, Lewysohn O, Cordova T, Ayalon D, Tsafriri A (1982). In vitro effects of cannabinoids on follicular function in the rat. Biol Reprod.

[68] Murphy SK, Itchon-Ramos N, Visco Z, Huang Z, Grenier C, Schrott R (2018). Cannabinoid exposure and altered DNA methylation in rat and human sperm. Epigenetics.

[69] Li Y, Wu Q, Li X, Von Tungeln LS, Beland FA, Petibone D (2022). In vitro effects of cannabidiol and its main metabolites in mouse and human Sertoli cells. Food Chem Toxicol.

[70] Holmes SD, Lipshultz LI, Smith RG (1983). Effect of Cannabinoids on human sertoli cell function in vitro. Arch Androl.

[71] Rajpert-De Meyts E, Hoei-Hansen CE (2007). From gonocytes to testicular cancer: the role of impaired gonadal development. Ann the N York Acad Sci.

[72] Rajpert-De Meyts E (2006). Developmental model for the pathogenesis of testicular carcinoma in situ: genetic and environmental aspects. Hum Reprod Update.

[73] Skakkebaek NE, Rajpert-De Meyts E, Buck Louis GM, Toppari J, Andersson AM, Eisenberg ML (2016). Male reproductive disorders and fertility trends: influences of environment and genetic susceptibility. Physiol Rev.

[74] Sharpe RM, Skakkebaek NE (2008). Testicular dysgenesis syndrome: mechanistic insights and potential new downstream effects. Fertil Steril.

[75] van den Driesche S, Kilcoyne KR, Wagner I, Rebourcet D, Boyle A, Mitchell R, et al. Experimentally induced testicular dysgenesis syndrome originates in the masculinization programming window. JCI Insight. 2017;2(6). Disponible sur: https://insight.jci.org/articles/view/91204. [Cité 14 Mars 2023]. 10.1172/jci.insight.91204 PMC5358493 28352662

[76] Welsh M, Saunders PTK, Fisken M, Scott HM, Hutchison GR, Smith LB (2008). Identification in rats of a programming window for reproductive tract masculinization, disruption of which leads to hypospadias and cryptorchidism. J Clin Invest.

[77] Scott HM, Mason JI, Sharpe RM (2009). Steroidogenesis in the fetal testis and its susceptibility to disruption by exogenous compounds. Endocr Rev.

[78] van den Driesche S, Kilcoyne KR, Wagner I, Rebourcet D, Boyle A, Mitchell R, et al. Experimentally induced testicular dysgenesis syndrome originates in the masculinization programming window. JCI Insight. 2017;2(6). Disponible sur: https://insight.jci.org/articles/view/91204. [Cité 15 juill 2022]. 10.1172/jci.insight.91204 PMC5358493 28352662

