# Supplementary figures and images for: Expression of the endocannabinoid system and response to cannabinoid components by the human fetal testis
Source: BMC Med. 2023 Jul 11;21:219. doi: 10.1186/s12916-023-02916-5 (PMC10334520; doi:10.1186/s12916-023-02916-5)

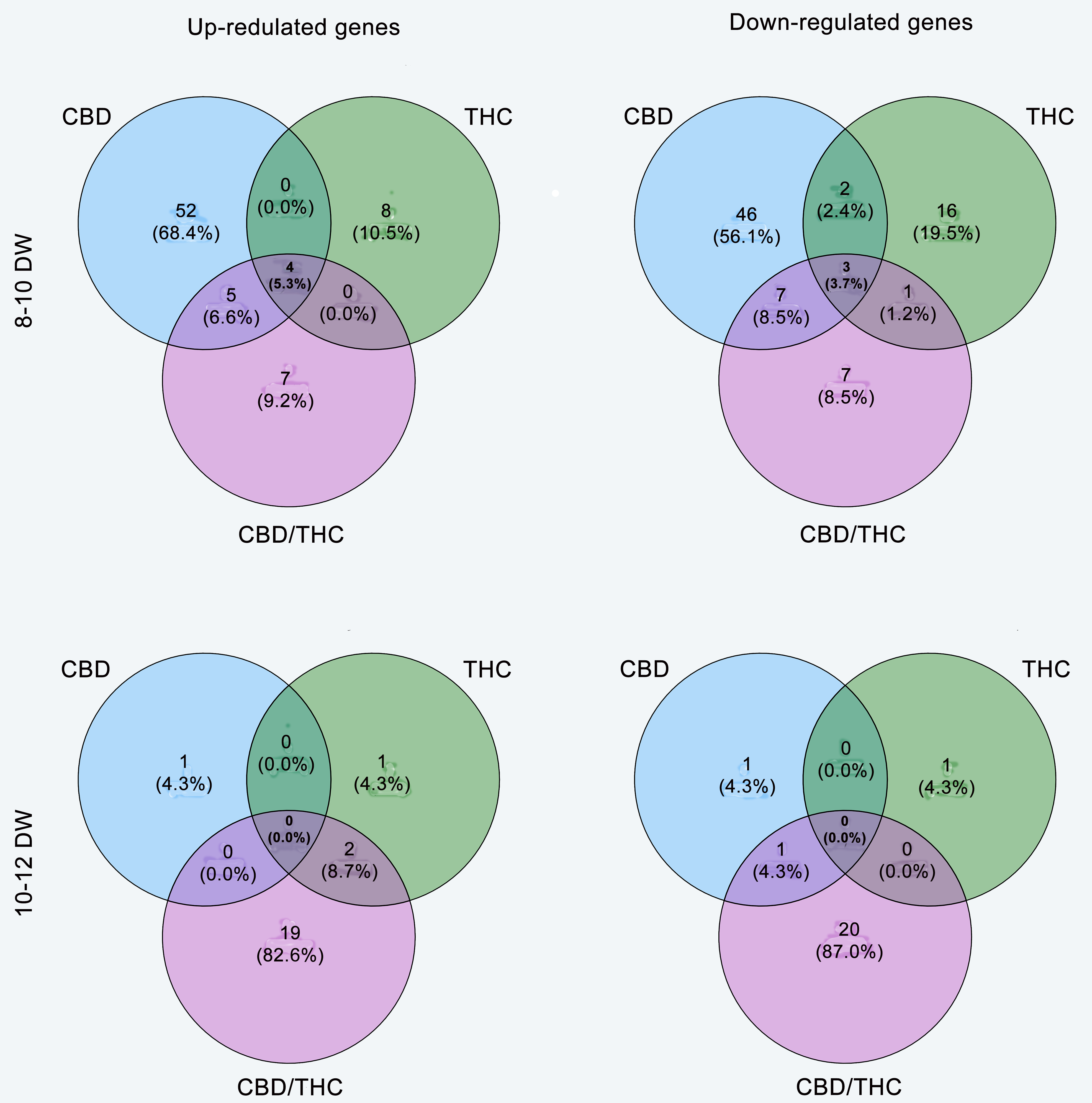

Supplement: Supplementary file 1 — Additional file 1: Figure 1. Venn diagrams representation of DEGs after CBD and/or THC treatments. [file 12916_2023_2916_MOESM1_ESM.tif]

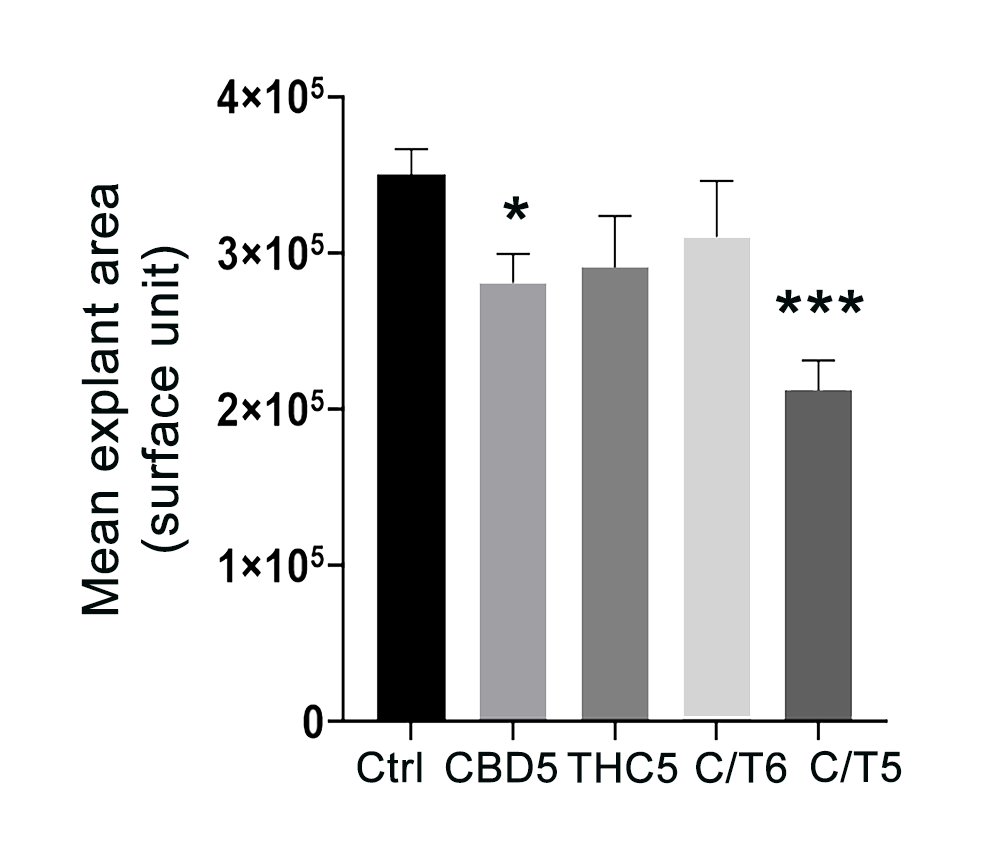

Supplement: Supplementary file 2 — Additional file 2: Figure 2. Mean area of fetal testis explants after culture with CBD and/or THC. Results are expressed related to the mean explant area. Data are means +/- SEM. Control and treated conditions were compared two by two using Wilcoxon tests. [file 12916_2023_2916_MOESM2_ESM.tif]
